# Drug Discovery of Plausible Lead Natural Compounds That Target the Insulin Signaling Pathway: Bioinformatics Approaches

**DOI:** 10.1155/2022/2832889

**Published:** 2022-03-20

**Authors:** Siba Shanak, Najlaa Bassalat, Ahmad Barghash, Sleman Kadan, Mahmoud Ardah, Hilal Zaid

**Affiliations:** ^1^Faculty of Sciences, Arab American University, P.O Box 240, Jenin, State of Palestine; ^2^Faculty of Medicine, Arab American University, P.O Box 240, Jenin, State of Palestine; ^3^Computer Science Department, German Jordanian University, Madaba Street. P.O. Box 35247, Amman 11180, Jordan; ^4^Qasemi Research Center, Al-Qasemi Academic College, P.O Box 124, Baqa El-Gharbia 30100, Israel

## Abstract

The growing smooth talk in the field of natural compounds is due to the ancient and current interest in herbal medicine and their potentially positive effects on health. Dozens of antidiabetic natural compounds were reported and tested in vivo, in silico, and in vitro. The role of these natural compounds, their actions on the insulin signaling pathway, and the stimulation of the glucose transporter-4 (GLUT4) insulin-responsive translocation to the plasma membrane (PM) are all crucial in the treatment of diabetes and insulin resistance. In this review, we collected and summarized a group of available in vivo and in vitro studies which targeted isolated phytochemicals with possible antidiabetic activity. Moreover, the in silico docking of natural compounds with some of the insulin signaling cascade key proteins is also summarized based on the current literature. In this review, hundreds of recent studies on pure natural compounds that alleviate type II diabetes mellitus (type II DM) were revised. We focused on natural compounds that could potentially regulate blood glucose and stimulate GLUT4 translocation through the phosphoinositide 3-kinase (PI3K)/protein kinase B (Akt) pathway. On attempt to point out potential new natural antidiabetic compounds, this review also focuses on natural ingredients that were shown to interact with proteins in the insulin signaling pathway in silico, regardless of their in vitro*/*in vivo antidiabetic activity. We invite interested researchers to test these compounds as potential novel type II DM drugs and explore their therapeutic mechanisms.

## 1. Main Text

Diabetes mellitus (DM) is described as a chronic disease that is characterized by progressive failure of the pancreatic *β*-cells. It occurs when the pancreas becomes unable to produce sufficient amounts of insulin (type I DM “here introduced as T1DM,” also known as insulin-dependent diabetes mellitus), when the body becomes unable to use the insulin it produces effectively (type II DM “here introduced as T2DM,” also known as adult onset diabetes mellitus, noninsulin-dependent diabetes mellitus), or during pregnancy (known as gestational diabetes mellitus, GDM) [1].

The WHO characterizes hyperglycemia that is identified during pregnancy for the first time as gestational diabetes mellitus (GDM). The amount of people with GDM is noticeably increasing worldwide. GDM is known to be the most common pregnancy metabolic disorder appearing in about 2–10% of all pregnant women who may recover from it after delivery. On the other hand, GDM is considered similar to the T2DM type of diabetes in several aspects including inadequate insulin responsiveness and secretion. Furthermore, about 5–10% of women with GDM found that they had T2DM after delivery. GDM is mentioned in our review, but it is further analyzed in the review by Santangalo and colleagues [2].

T1DM is the form of diabetes mellitus which might exist as a result of the autoimmune destruction of the pancreatic beta-cells that produce insulin [3]. Symptoms of this form of diabetes include constant hunger, vision changes, polyuria, polydipsia, fatigue, and weight loss. In T1DM, the pancreas does not produce sufficient amount of insulin. Thus, insulin therapy is needed. This form of the disease is not widespread, where only 5–10% of patients with diabetes have T1DM. However, T1DM in most countries is known to be the most common chronic disease effecting young individuals of age under 18 [4]. HLA genotypes along with many genes were analyzed to prove that T1DM is partly inherited and that its pathogenesis is complex and multifactorial. On the other hand, the escalated incidence of T1DM is one indicator for modern life styles [5], including an imbalanced diet, e.g., vitamin D3 deficiency or viral infection [6, 7]. Most probably, the disease is associated with the autoimmune processes that lead to autoantibodies destroying beta-cells of the pancreas, what leads to insulin scarcity and eventually the damage of the organ [5].

In T2DM (the noninsulin-dependent diabetes mellitus), the body normally produces insulin, but cells are not sensitive to it the way they should. T2DM is commonly caused by a sequence of environmental and hereditary factors, including mainly population growth, obesity, aging, lack of physical activity, and urbanization. As a result, T2DM is widely spread, where it represents nearly 90% of all diabetes cases. About 6% of the adult population in developed countries suffers from T2DM [8]. According to the 2014 report of the World Health Organization (WHO), 9% of adults of age 18 and older had diabetes. Additionally, diabetes was the direct cause of 1.5 million deaths in 2012. About 425 million people worldwide are diabetic nowadays, and the relevance is expected to increase by 48% in 2045 [9].

T2DM is described as a chronic metabolic disease. One major complication of T2DM is hyperglycemia, which may result in many other classical symptoms such as polyphagia, polydipsia, polyuria, and extraordinary glucagon secretion [10, 11]. The rate of appearance of complications and their severity are directly proportional to the degree of hyperglycemia [12, 13]. At a later stage, hyperglycemia might result in a serious damage in the nerves and the blood vessels in addition to irritability and blurred vision. Moreover, if diabetes is left without treatment, it can result in a group of serious complications that includes kidney failure, impotence, heart disorders, foot ulcer, eyes damage, strokes, ketoacidosis, and death [14].

Next stages of the disease eventually lead to hyperglycemia and hyperinsulinemia as the circulating blood glucose increases which pressures the beta-cells of the pancreas to secrete extraordinary amounts of insulin. Consequently, such actions will lead to damage of the pancreas. On the other hand, insufficient amounts of insulin develop comparable symptoms such as in T1DM, although they are less noticed. In such specific cases, insulin should be used to treat diabetic patients. In this scenario, “noninsulin-dependent diabetes mellitus” (NIDDM) is not valid term anymore. Moreover, T2DM is not nowadays referred to “adult-onset diabetes” as this term is not accurate anymore since it describes diabetes instances in young people that might have resulted from modern life style, with excessive food intake leading to extra bodyweight with insufficient exercise periods [11]. Indeed, the numbers of young people affected with T2DM are increasing alarmingly and make up a great portion of the cases known to have T2DM worldwide. The age limit is not a standard in defining this disease anymore.

Insulin hormone is secreted by beta-pancreatic cells as a response to the increase of glucose in blood, and it maintains the homeostasis of blood glucose. Insulin mediates this process in two ways: the first is by limiting the liver production and release of glucose and the other is via increasing the rate of glucose uptake in the adipose tissues, liver, and skeletal muscle [15, 16]. A progressive decrease in insulin production as an ultimate failure of pancreatic *β*-cell function might later lead to glucose intolerance and at a later stage to T2DM that complicates over time [17]. Moreover, the pathogenesis of T2DM is highly effected by the mass reduction of pancreatic *β*-cells [15, 18]. Indeed, in T2DM, the pancreatic *β*-cell mass is negatively affected by dyslipidemia and hyperglycemia because they noticeably increase pancreatic *β*-cell apoptosis and necrosis as presented in results of rodent models of the disease and in cultured rodent and human islet cells [19–21].

T2DM can be prevented or treated by changing the life style, avoiding obesity, avoiding tobacco, and increasing exercise periods. In some T2DM patients, weight loss even through surgery appeared to be an effective treatment in some cases. If all previous treatments were insufficient for specific cases, medications including or excluding insulin might be prescribed. On the other hand, lowering the risk of diabetes and predisposing factors can be achieved by following healthy diets including foods with high content of phytochemicals and high antioxidant capacities presented in some epidemiological investigations [22]. *Caragana intermedia* plant is a traditional successful treatment where physicians and practitioners have used known herbs, medicinal plants, and food consumption control for treating some symptoms of diabetes. More details are in the section “Natural Compounds Affecting the Insulin Signaling Pathway.”

## 2. Glucose Uptake into Cells

**Figure 1:**
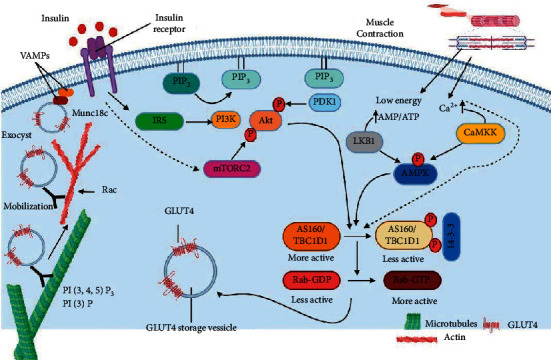
Signaling pathways for insulin and contraction-stimulated GLUT4 translocation into muscle PM.

In mammalian cells, glucose can be considered as the key metabolic fuel [23]. The cellular metabolism of glucose depends on its ability to penetrate through the membrane into cells. Glucose transport is mediated by specific carriers that allow access and exit of glucose into and from cells. There are two well-studied families of glucose transporters which are the sodium-glucose linked transporters (SGLT) and main glucose transporters (GLUTs). SGLTs are a family of glucose transporters, which depends on sodium gradient as a driving force for glucose transport. Six transporters of this family are known for now; SGLT1 and SGLT2 are the main transporters, which act as cotransporters among this group. These transporters are mainly in gastrointestinal epithelium of the kidneys and nephron [24]. GLUTs, the second form of transporters, are independent of sodium and act by accelerated diffusion. Fourteen transporters of this family are known for now, and they have similar structures, but differ in their affinity to sugars and their dispersion in the various body tissues [25]. GLUT1-5s are the central transporters known in this family. GLUT1 is the most common transporter in the body tissues, and it is controlling the basic absorption of glucose in the cells. GLUT2 has low affinity for glucose and is mainly located in the pancreatic beta-cells, liver, and kidney. It is important for glucose sensing in the beta-pancreatic cells and has a role in renal glucose reabsorption. GLUT3 is mainly found in neurons and has high affinity to glucose [24, 26]. GLUT4 is expressed in tissues labelled as insulin sensitive, particularly the skeletal muscle, liver, and adipose tissue. GLUT4 reciprocally circulates between the plasma membrane and intracellularly. GLUT4 is noticeably compartmentalized in the vesicles of intracellular membrane which explains having no access to the extracellular space specifically under basal conditions. Upon insulin stimulation (and muscle contraction), GLUT4 controls the translocation to cell surface from intracellular compartments. GLUT4 is responsible for translocating glucose into the cell specifically from the extracellular milieu. The significant role of GLUT4 in insulin-regulated uptake of glucose labels GLUT4 as an important member of the normal glucose homeostasis process and consequently as a key player in T2DM type of diabetes and insulin resistance. Indeed, GLUT4 is the main transporter implicated in decreasing glucose levels in blood after its appearance. Because of the abovementioned factors, GLUT4 is thoroughly researched in diabetic models [11, 24, 26, 27]. Insulin and physical activity stimulate signaling cascades that ultimately lead to GLUT4 transportation to the plasma membrane via activating insulin receptor and the downstream pathway as well as the AMPK pathway as described in [11] and in Figure 1.

## 3. Insulin Signaling

Insulin balances the metabolism of carbohydrates, fats, and proteins through the insulin signaling cascade, which associates PI3K, Akt1, Akt2, insulin receptor (IR), insulin receptor substrate (IRS), and others [16]. IR is one receptor of the tyrosine kinases family which includes also the insulin-like growth factor-I receptor (IGF-IR), which binds its receptor IGF-I and activates PI3K, and also the insulin receptor-related receptor (IRR), an alkali sensor that calibrates the metabolic bicarbonate excess [10]. Such receptors are composed of two extracellular-subunits (135 KDa) in addition to other two transmembrane subunits (95 KDa) [28]. At the time when insulin binds to its receptor, autophosphorylation of this receptor specifically on the *β* subunit on the tyrosine residues of (Tyr1158, Tyr1162, and Tyr1163) takes place.

Many of the insulin receptor substrates along with many other substrates have confirmed roles in mediating the binding of their effectors, which are known to contain the Src homology domain 2 (SH2). Examples of these substrates include the GRB2-associated-binding protein 1 (Gab-1), the adapter protein containing PH and SH2 domain (APS), the docking protein P62dok, the E3 ubiquitin-protein ligase Cbl, and the SHC-transforming protein (SHC) in addition to the main insulin receptor substrates IRS-1, IRS-2, IRS-3, and IRS-4. This is a different behavior compared to the other receptor tyrosine kinases which bind to their effectors directly [29, 30]. Present, there is no doubt that tyrosine phosphorylation activates IR and IRS proteins, while serine phosphorylation and protein tyrosine phosphatases (PTPs) lead to their inhibition [30]. Moreover, IR and IR-serine phosphorylation results in the reduction of the tyrosine phosphorylation which promotes their interaction with 14-3-3 proteins (the name refers to the elution pattern on DEAE chromatography (14^th^ fraction) and the fraction number (3.3) in a later purification step of the gel electrophoresis), a family of conserved regulatory proteins that activate tyrosine and tryptophan hydroxylases, which are involved in the biosynthesis of neurotransmitters [31]. Furthermore, the reduction of the tyrosine phosphorylation is reported to impair the insulin-stimulated signaling [32]. Additionally, insulin signaling can be attenuated as a result of the accelerated dephosphorylation of tyrosine by PTPs. On the other hand, the tyrosine phosphorylation of IR and IRS-1 in muscles can escalate via a knockout of a cytoplasmic PTP protein that is tyrosine phosphatase 1B (PTP1B), which eventually increases the insulin sensitivity [33].

The combination of IRSs with p85, the regulatory subunit of PI3K [34], causes the enrollment of the PI3K catalytic subunit, p110. The p110 phosphorylates phosphatidylinositol (4,5) bisphosphate (PtdIns (4,5) P2) to generate PtdIns (3,4,5) P3 (PIP3) [35]. The persistent increment of PIP3 causes the enlistment of the related main player of insulin signaling, Akt, that acts from cytosol directly to the plasma membrane through the process where it binds exactly a pleckstrin homology (PH) domain existing at Akt amino terminus [36]. Such combination makes Akt in action similar to phosphoinositide-dependent kinase-1 (PDK1) and mTOR complex 2 (mTORC2) that has a function similar to pyruvate dehydrogenase kinase 2 (PDK2), which leads to phosphorylating Akt specifically at thr308 and ser473 [37]. Once Akt is activated, it separates from the plasma membrane and plays a significant role in the regulation of the insulin-dependent processes directly after it gets phosphorylated by numerous substrates [35]. Moreover, Akt causes phosphorylation and later the inhibition of glycogen synthase kinase 3 (GSK-3) [16]. Such a process leads to glycogen synthase activation that has a catalyzation role in glycogen synthesis final steps [35]. Furthermore, the Rab-GTPase-activating protein and Akt substrate of 160 KDa (AS160) are also phosphorylated and inhibited by Akt [38]. These events eventually trigger the GLUT4 movement to PM specifically from the intracellular compartments, what leads to Rab small GTPases activation and consequently regulating the reorganization of the cytoskeleton [36]. Similarly, some transcription factors such as few of the winged helix or fork head box protein O (FOXO) class of transcription factors are also phosphorylated by Akt. Such transcription factors have significant roles in the expression of gluconeogenic and lipogenic enzymes. For instance, FOXO1 triggers the activation of the liver gluconeogenic genes [39] where it also inhibits adipogenesis [39].

Phosphatase and tensin homolog (PTEN) has a significant role in insulin signaling and translocation of GLUT4. PTEN dephosphorylates the third hydroxyl position of the phosphoinositides inositol ring, especially PIP3. Additionally, it is reported to be a PI3K-Akt signaling negative regulator and a significant key factor in insulin signaling. Moreover, the basal and insulin-stimulated PIP3 levels have increased due to the microinjection of anti-PTEN antibody, which eventually leads to the accelerated GLUT4 translocation to PM [11].

Peroxisome proliferator-activated receptors (PPARs) are members of the superfamily of nuclear receptors (NRs), which control the transcription of several genes. In specific, the activation of PPAR*γ* in mature adipocytes modulates the expression/phosphorylation of a number of genes encoding proteins crucial in various steps of the insulin signaling pathway. For example, the treatment with PPAR*γ* agonist was found to increase the tyrosine phosphorylation of the insulin receptor (IR) and IRS-1 and to induce activation of Akt/PKB. Indeed, an enhancement in insulin-stimulated activity of PI 3-kinase and Akt as well as an augmented Akt phosphorylation in skeletal muscles were noticed [40].

Dipeptidyl-peptidase 4 (DPP4) is a glycoprotein exopeptidase (110 kDa) abundantly expressed throughout the body. A wide range of substrates for this protein were found to induce insulin secretion. Incretin hormone is a substrate that binds to the beta-pancreatic cells, attenuates glucagon release, and induces insulin secretion. Two targets are glucagon-like peptide 1 (GLP1) and GIP which simulate glucagon in their actions, where DPP4 inhibition co-occurs with their activation [41]. Stromal cell-derived factor-1*α*/CXCL12 is a substrate that activates Akt signaling and induces the survival of the pancreatic beta-cells [41]. Substance P interacts with proteins that aid in phosphorylation and consequent inhibition of IRS-1 [42]. Shaikh and colleagues reviewed that several natural plants were found to inhibit DPP4, and the natural phytochemicals include a set of alkaloids, flavonoids terpenoids, phenols, and glycosides [43].

The detailed insulin signaling pathway is beyond the scope of this review, and the authors are directed to excellent reviews in this topic.

## 4. Natural Compounds Affecting the Insulin Signaling Pathway

The homeostasis of glucose relays mainly on the activity of the pancreas, skeletal muscle, adipocytes, and liver. Insulin is required to maintain glycogenolysis and hepatic gluconeogenesis, as well as the disposal of peripheral glucose, in a balanced state. The resistance of insulin or inefficiency, as well as glucose metabolism disorders, causes elevated blood sugar levels in diabetic patients [44]. Therapies by natural medicine could increase the uptake of glucose into skeletal muscle cells and adipocytes through translocation activation of the glucose transporter type 4 (GLUT4) directly to the cell PM and leading to escalation in the uptake of glucose. GLUT4 is predominantly present through the adipose tissues in human, as well as the cardiac and skeletal muscles [45]. Insulin promotes glucose transfer by translocating GLUT4 to the PM in muscles, adipocytes, and liver [46]. The process of activating PI3K/Akt and AMPK in GLUT4 is typically powered by the urge to increase the utilization of insulin-dependent glucose. The pathway of PI3K/Akt stimulates a number of downstream enzymes to speed up GLUT4 translocation. On the other hand, the AMPK pathway, which functions as an energy sensor, boosts the translocation of GLUT4 by activating glycolysis and ATP-dependent *β*-oxidation [47, 48]. The pathways of AMPK and PI3K/Akt play a rule to control the inhibition of the hepatic gluconeogenesis by interfering with the expression of some of the main process enzymes such as phosphoenolpyruvate carboxykinase (PEPCK) in addition to the glucose 6-phosphatase (G6Pase) [49–52]. Peroxisome proliferator-activated receptor gamma (PPAR*γ*) controls the glucose metabolism, lipid uptake, and adipocyte differentiation. The expressions of GLUT4 and insulin sensitivity marker adiponectin (AdipoQ) are both maintained via PPAR*γ* [53]. The beta-cell dysfunction and insulin resistance are the main characterizations of type 2 diabetes mellitus (T2DM). A compensatory rise in the secretion of insulin occurs at first, maintaining the levels of glucose in the standard range. Beta-pancreatic cells shift as the disease progresses, and insulin secretion is unable to preserve glucose homeostasis, resulting in hyperglycemia [54]. Research groups universally study the efficiency of natural compounds in treating diabetes [55, 56]. Alkaloids [57], glycosides [58], polyphenols [59], carotenoids [60], terpenoids [60], flavonoids [61], anthocyanins [62], tocopherol [63], peptidoglycans [64], steroids [65], saponins [66], xanthones [67], and polysaccharides [68] have been presented as antidiabetic properties. Forty-two natural products were analyzed for their potency to treat diabetes by lowering in vivo blood glucose levels and activating in vitro GLUT4 translocation (Table 1). These compounds were tested for having a role with AMPK, G6Pase, PI3K/Akt, GLUT, PPAR*γ*, or PEPCK signaling specifically in the liver, skeletal muscle, and pancreas (Table 1).

Rutin is a flavonoid glycoside that occurs naturally in many fruits and vegetables. It alters glycolytic and gluconeogenic enzymes, thus enhancing glucose homeostasis. It has stimulatory effects on glucose absorption as well [114]. In rats and specifically the soleus muscle, rutin improved glucose uptake. These findings indicate that rutin increased the uptake of glucose in the soleus muscle of rats by activating phosphoinositide 3-kinase (PI3K), mitogen-activated protein kinase (MAPK), and typical protein kinase C pathways. Moreover, rutin affects glucose uptake in a way that mimics the role of insulin in maintaining glucose homeostasis [115]. Nonetheless, the rutin effect on the uptake of glucose was found to be blocked after a planned treatment with cycloheximide, an inhibitor of protein synthesis, wortmannin, an inhibitor of PI3K, colchicine, a depolymerizing agent of microtubules, HNMPA(AM)3, an inhibitor for the insulin receptor tyrosine kinase, RO318220, an inhibitor of protein kinase C, and PD98059, an inhibitor of mitogen-activated protein kinase kinase (MEK). This suggests the involvement of these pathways in affecting glucose uptake [115].

Morin is a pentahydroxyflavone that is known to as a trigger and a sensitizer of insulin receptor which helps stimulating metabolic pathways. Morin is a PTP1B noncompetitive inhibitor with *K*_i_ that is within the *μ*M range. It increases phosphorylation of the insulin receptor (IR) and protein kinase B (Akt) in HepG2 cells, inhibits gluconeogenesis, and increases the synthesis of glycogen [70]. Moreover, in a diabetic mouse model, it is reported that morin activates the Akt/eNOS pathway and improves the endothelial dysfunction [116].

Gallotannins are capable of increasing the activity of glucose-6-phosphatase (G6Pase) while decreasing glucokinase (GK) activity, thus inducing beta-cells in the pancreas and leading to insulin release. The mRNA expression of PI3K and GLUT4 as well as the phosphorylation of insulin receptor substrate-1 (IRS-1) and IR were increased in L6 muscle cells treated with gallotannins [71].

On the other hand, gallic acid is reported to lower blood glucose in diabetic rats [117], where it also improves the uptake of glucose by compartmentalizing GLUT4 to the PM in adipocytes specifically isolated from STZ-treated rats via activating the signaling pathway PI3K [72].

Oleanolic acid is a pentacyclic triterpenoid found in a variety of vegetables, fruits, and herbs in nature. IR phosphorylation was significantly increased in CHO/hIR cells treated with oleanolic acid. Concomitantly, it is also augmented in glucose uptake presence and absence in L6 myotubes [73].

Mangiferin enhances the expression of GLUT4 protein and its subsequent translocation to 3T3-adipocytes and the L6 myocytes surface, resulting in an increased glucose uptake by the cells [74].

Berberine, arecoline, and vanillic acid could augment glucose uptake in 3T3-L1 adipocytes up to three folds at a micromolar range. Berberine as well as vanillic acid could significantly increase the translocation of GLUT4 via the AMPK-dependent pathway. The same effect of arecoline was exerted via the PPAR*γ* pathway. These phytochemicals might also help in preventing some secondary complications of diabetes as they could significantly reduce enzymes expression of the proteins involved in cholesterol and fatty acid synthesis [75].

3*β*-Taraxerol could lead to insulin-stimulated uptake of glucose via the translocation and activation of the glucose transporter (GLUT4) taking place in the IR and PI3K-dependent pathway. The destiny of glucose after the insulin-stimulated uptake of glucose was determined using a synthesis assay of glycogen involving GSK3 beta-suppression and PKB activation [76].

Astragalus polysaccharide therapy could partially restore insulin-induced protein kinase B-phosphorylation of ser473 and translocation of GLUT4 in the skeletal muscles of diabetic KKAy mice, suggesting that *Astragalus* can possibly act as an insulin sensitizer in the type 2 diabetes treatment [77].

Cyanidin-3-O-*β*-glucoside and protocatechuic acid in human omental adipocytes and 3T3-L1 cells have insulin-like activity. Moreover, PPAR*γ* activity was boosted in cells treated with 50 *µ*M cyanidin-3-O-*β*-glucoside and 100 *µ*M protocatechuic acid and ultimately enhanced translocation of GLUT4 and secretion of adiponectin, leading to incrementing glucose uptake [78].

Similarly, daidzein is one of the compounds reported to enhance the uptake of glucose. Western blotting studies in L6 myotubes in the insulin absence showed that daidzein enhances AMPK phosphorylation and translocation of GLUT4 in L6 myoblasts that are transfected with a GLUT4 cDNA-coding vector [79].

Iridoid, catalpol, specioside, and verminoside were found to enhance the translocation of GLUT4 to the cell surface in skeletal muscle cells from intracellular compartments, without compromising cell viability. An antibody-coupled colorimetric assay was used to determine the amount of GLUT4 on the cell surface of nonpermeabilized L6-GLUT4myc myotubes [80].

Lupeol and lupeol-trifluoroacetate showed substantial stimulation of glucose uptake in L6 cells, which was linked to increased translocation of GLUT4 and activating the IRS-1/PI3K/Akt-dependent signaling pathway. The integrity of *α*, *β*-unsaturated carbonyl and acetyl moieties was important in retention of the stimulatory effect on the uptake of glucose, according to a structure-activity relationship analysis of these analogs [81].

Palmitic acid (PA) rapidly mediated translocation of GLUT4 and enhanced the uptake of glucose in the rat skeletal muscle cell line L6 according to immunofluorescence findings. In a time-dependent manner, PA increased phosphorylation of Akt, AMPK, and extracellular signal-related kinase1/2 (ERK1/2). Moreover, bound PA on the cell surface could cause Akt phosphorylation [82]. In rat adipocytes, PA also increased basal and insulin-stimulated glucose incorporation by three-fold and two-fold, respectively. The capacity of PA to promote glucose absorption was additive to insulin-induced stimulation and proportional to PA concentrations between 0.15 and 2.40 mM [118].

*α*, *β*-Amyrin is reported to notably suppress the differentiation of adipocytes via lowering the levels of expression of adipogenesis-related main transcription factors, such as PPAR*γ*. Furthermore, the expression of GLUT4 was significantly higher in 3T3-L1 adipocytes treated when with *α*, *β*-amyrin. This suggests that *α*, *β*-amyrin enhances the uptake of the glucose and carbohydrate metabolism [83].

Ursolic acid could promote glucose absorption via the PI3K pathway. The differentiated 3T3-L1 adipocytes were initially pretreated with the inhibitors AMPK, MAPK, and PI3K. Next, 10 *µ*M of ursolic acid in the presence or absence of 1 *µ*g/mL insulin was used to treat the cells. Later, wortmannin which is a known inhibitor for PI3K was used with a concentration of 1 *µ*M leading to blocking insulin-stimulated uptake of glucose while having only a slight effect at rest. In basal or insulin-stimulated states, ursolic acid-stimulated uptake of glucose was not affected by the AMPK inhibitor. Moreover, in basal and insulin-stimulated states after a 24-hour incubation time, it was discovered that 10 *µ*M ursolic acid elevated GLUT4 translocation to the cell membrane as well as increased the total cellular GLUT4 expression [84]. It also improved glucose intolerance by activating IRS-PI3K-Akt-dependent signaling pathways to induce the translocation of GLUT4 and through increasing insulin receptor expression. A combination treatment of ursolic acid and rosiglitazone alleviated high-fat diet-induced glucose sensitivity and insulin resistance in C57/BL/6J mice, by raising the homeostatic model evaluation index [85] of protocatechuic acid (4-hydroxybenzoic acid) increased GLUT4 translocation and the uptake of glucose in adipocytes of human by activating the insulin signaling pathway, via increasing the phosphorylation IRS-1 tyrosine (by 40% compared to vehicle) and through inducing downstream events, such as PI3K binding to IRS-1 and Akt phosphorylation (+100% and +180%, respectively, compared to vehicle). The insulin-like activity of protocatechuic acid seems to be regulated by IR, as these effects were completely eliminated when autophosphorylation of insulin receptor was inhibited [86].

Oral administration of myo-inositol of 1 g/kg bodyweight (BW) glucose to C57BL mice exactly 30 minutes before 2 g glucose/kg BW postoral injection leads to increased translocation of GLUT4 in skeletal muscles as well as lowered plasma glucose and insulin levels [87]. As a positive effector on insulin-resistant tissues including polycystic ovary syndrome-endometrium, myo-inositol may be a possible insulin sensitizer, triggering the activation of AMPK and increasing GLUT4 levels, which consequently increase the glucose absorption in the endometrial cells of human [88].

When diabetic rats were treated with naringenin (25 mg/kg BW) for 45 days, hyperglycemia and hyperinsulinemia were reduced, a lipid profile was restored, a membrane lipid peroxidation was reduced, an antioxidant activity was increased, and hepatic function markers were improved. In diabetic rats, naringenin therapy modulated expression of TNF-*α* and GLUT4, restored the histological abnormalities, and increased insulin sensitivity. As a result, glucose homeostasis could be restored [89].

Analyzing the skin of chum salmon (*Oncorhynchus keta*), some oligopeptide compounds can be acquired including marine collagen peptides that are normally enzymatically hydrolyzed. Marine collagen peptides at a high concentration (4.5 g/kg/day) can increase insulin sensitivity in the diabetic rats by upregulating the expression of GLUT4 while decreasing the expression of inflammatory cytokines, oxidative stress biomarkers, and adipocytokines. Moreover, they enhance the glucose metabolism and insulin sensitivity [90].

Bavachin could activate the adipogenic transcriptional factors proliferator-activated receptor (PPAR*γ*) in addition to CCAAT/enhancer binding protein (C/EBP). In adipocytes, Bavachin enhanced the expression and secretion of fAdiponectin. It also increased the insulin-induced uptake of glucose in differentiated adipocytes and myoblasts. Moreover, bavachin increased the uptake of glucose through augmenting translocation of GLUT4 to the plasma membrane in differentiated adipocytes by activating AMPK and Akt pathways both in insulin presence and absence [91].

Rosmarinic acid could enhance GLUT4 expression in skeletal muscles of both the STZ-induced diabetic rats and HFD diabetic rats [92].

Dehydroeburicoic acid is a triterpenoid compound found in *Antrodia camphorata*. Treatment of mice with dehydroeburicoic acid could decrease HFD-fed mediated hyperglycemia, hyperinsulinemia, hyperleptinemia, hypercholesterolemia, and hypertriglyceridemia. Additionally, the membrane GLUT4 levels were increased. Phospho-Akt at different concentrations and AMPK phosphorylation were also enhanced in hepatic and skeletal muscles in mice. The levels of mRNA in carnitine palmitoyl transferase Ia (CPT-1a) and hepatic fatty acid oxidation enzymes such as PPAR*α* were also found to be augmented [93].

Baicalin and its metabolites were shown to enhance glucose consumption, which could be linked to the inhibition of main gluconeogenic genes such as glucose-6-phosphatase (G6Pase), glucose transporter 2 (GLUT2), and phosphoenolypyruvate carboxykinase (PEPCK). In insulin-resistant HepG2 cells, baicalin and its three metabolites could downregulate gluconeogenic genes and GLUT2, via the PI3K/Akt signaling or AMPK pathways [94].

Kazinol B could increase intracellular lipid aggregation, induce the gene expression of PPAR*γ* and CCAAT/enhancer binding protein-alpha (C/EBP), enhance the transcriptional activation of PPAR*γ*, and augment the uptake of 2-[N-(7-nitrobenz-2-oxa-1,3-diazol-4-yl)amino]-2-deoxy-d-glucose (2-NBDG) in 3T3-L1adipocytes cells by upregulating gene expression and translocation of GLUT4. Furthermore, it could improve adiponectin gene expression and secretion, which is related to a decreased risk of T1DM and T2DM types of diabetes [95].

One of the tannin forms isolated from *Ishige foliacea* is octaphlorethol A. It inhibits G6Pase and PEPCK activities in the liver and effects of the GLUT4-mediated uptake of glucose in skeletal muscle by activating AMPK which lead to suppressing gluconeogenesis. In C57BL/KsJ-db/db mice, the expression of GLUT4 was noticeably higher in the octaphlorethol A-treated group relative to control db/db mice group [96].

Phloridzin enhanced the expression of GLUT2, IR, G6Pase, IRS, and PEPCK in the liver tissue of STZ-and HFD-induced type II diabetic mice [97].

Pterosin A could reverse the elevated insulin resistance and serum insulin, inverse the decreased Akt and AMPK phosphorylation in muscles, upset the reduced muscle GLUT4 translocation, and override the elevated PEPCK expression in liver in dexamethasone-treated diabetic mice and db/db mice. Moreover, pterosin A played a key role in enhancing glucose uptake and AMPK phosphorylation in cultured human muscle cells. Furthermore, pterosin A inhibited the expression of inducer-enhanced PEPCK, while it activated acetyl-CoA carboxylase, AMPK, and phosphorylation of glycogen synthase kinase-3. Additionally, it decreased phosphorylation of glycogen synthase and increased intracellular glycogen levels in cultured liver cells [98].

Piceatannol is a metabolite and natural analog of resveratrol, a well-known AMPK activator. Western blotting studies in L6 myotubes in insulin absence revealed that piceatannol promotes GLUT4 translocation, glucose absorption, and phosphorylation of AMPK. In L6 myoblasts transfected with a GLUT4 cDNA-coding vector, piceatannol promoted translocation of GLUT4 to the plasma membrane as well as the uptake of glucose, as determined by immunocytochemistry. Moreover, piceatannol reduced blood glucose levels in the early stages and increased impaired glucose tolerance in the late stages of diabetes in db/db mice [99].

Resveratrol (0.005% and 0.02%, w/w), administrated to C57BL/KsJ-db/db mice, significantly decreased blood glucose, plasma free fatty acid, and triglyceride. After resveratrol supplementation, AMPK and downstream targets were activated, resulting in hepatic gluconeogenic enzyme activity, lower levels of blood HbA1c, and hepatic glycogen. On the other hand, skeletal muscle GLUT4 protein, pancreatic insulin protein, and plasma insulin levels were higher relative to control [102].

Chlorogenic acid enhanced IRS-1-PI3K-Akt activation and ultimately GLUT4 translocation in L6 myotubes [104]. Chronic administration of chlorogenic acid to Lepr (db/db) mice improved insulin sensitivity, glucose tolerance, dyslipidemia, and fasting glucose levels by inhibiting expression and activity of hepatic G6Pase, enhancing lipid profiles, attenuating hepatic steatosis, and increasing the uptake of glucose in skeletal muscles. Chlorogenic acid could additionally trigger AMPK, which resulted in beneficial metabolic effects including reduced fatty acid synthesis and hepatic glucose production [119].

Honokiol administrated orally to T2DM type diabetic mice, at 200 mg/kg dose, could substantially lower fasting blood glucose. In the liver, skeletal muscle, and adipose tissues of honokiol-treated mice, phosphorylation of downstream insulin signaling factors (such as Akt and ERK1/2) and IR*β* increased noticeably. Furthermore, in a dose-dependent manner in C2C12 myotube cells, honokiol increased insulin-stimulated phosphorylation of ERK1/2, Akt, and IR*β*. Honokiol could additionally increase insulin-stimulated translocation of GLUT4. Honokiol showed reversible competitive inhibitory activity against PTP1B with strong selectivity in the in vitro and in vivo studies [106].

Kaempferol (0.05% in the diet) administrated to middle-aged obese HFD-induced mice significantly improved circulating lipid profile, hyperinsulinemia, and hyperglycemia. As a result, an improved peripheral insulin sensitivity was noticed. Treatment with kaempferol reversed the effects of high-fat diet on GLUT4 and the expression of AMPK in the adipose and muscle tissues of obese mice. In skeletal muscle cells, kaempferol increased lipolysis and prevented glucose absorption, GLUT4 expression, AMPK activity, and glycogen synthesis in high fatty acid-impaired cells [108].

3-Bromo-4,5-bis(2,3-dibromo-4,5-dihydroxybenzyl)-1,2-benzenediol (CYC31) is a PTP1B inhibitor. It is isolated from the red algae *Rhodomela confervoides*. In C2C12 myotubes, CYC31 increased insulin signaling activity and promoted the uptake 2-NBDG through translocation of GLUT4 [109].

One of the sesquiterpenoids derived from *Caragana intermedia* plant is carainterol A. It could increase IRS-1 protein levels and phosphorylation of downstream protein kinase Akt in HepG2 cells at low levels of micromolar concentrations [110].

Bis(2,3-dibromo-4,5dihydroxybenzyl) ether (BDDE), a PTP1B inhibitor, has been discovered in a novel bromophenol isolated from red alga. In HepG2 cells, insulin-resistant glucose uptake was increased. BDDE also inhibited PTP1B expression when activating insulin signaling substrates and downstream signals including IR*β*, Akt, IRS-1/2, and PI3K [110].

Galangin is a natural inhibitor of DPP4. Galangin induced reduced levels of glucose in skeletal muscles at a higher level than when the cells were treated alone with insulin, what makes it a promising drug for the treatment of diabetes [112].

Chrysin is another natural inhibitor of DPP4. Glucose uptake levels in skeletal muscles were studied by Kalhotra and colleagues in treatments including insulin alone or in combination with chrysin on differentiated skeletal muscle cells. Combination treatment was found to be not toxic to the skeletal muscles, and it was found to augment glucose uptake by the skeletal muscles [113].

It is appreciated that most of the abovementioned phytochemicals enhance GLUT4 translocation and activity via the insulin signaling pathway in most cases. These results indicate that the putative upcoming natural antidiabetic drug is thought to be glucose space and GLUT4 translocation enhancer.

## 5. Docking Experiments Investigating Plausible Natural Effector Ligands That Bind to the Protein Targets of Insulin Signaling

With the progress in research, bioinformatics and chemoinformatics methods have enabled more throughput screening on natural products that originate from plants, fungi, and other natural origins. The health benefits that these natural products provide target the treatment of various ailments. One major focus of studies with natural products for diabetes treatment (particularly through molecular docking) is on those ligands that offer effectors for proteins involved in insulin signaling. We introduce the major docking studies conducted so far for the natural products that interact with protein hubs in insulin signaling. Structural evidences for resolved major proteins involved in the insulin signaling cascade are detailed in Supplementary Material.

### 5.1. Insulin Receptor and PTP-1*β*

Recently, 43 phytochemicals were extracted from the medicinal plants *Ficus racemosa*, *Thespesia populnea*, *Ficus lacor* Buch.-Ham, *Ficus benghalensis*, and *Ficus religiosa* [120]. The phytochemicals were screened for their interaction profile with the target proteins that serve as hubs in diabetes. The three target proteins include mono-ADP ribosyltransferase-sirtuin-6 (SIRT6), aldose reductase (AR), and insulin receptor (IR). SIRT6 indirectly affects insulin signaling, as SIRT6 deficiency in mice resulted in increased glucose uptake, activated insulin signaling, and augmented Akt phosphorylation [121]. AR is responsible for the reduction of glucose to sorbitol, an inactive alcohol, in the polyol pathway in an NADPH-dependent manner. This pathway normally utilizes small amounts of glucose. However, under hyperglycemic conditions, sorbitol accumulates and NADPH drastically decreases [122]. For the aim of this review, we concentrate here on work conducted for insulin receptor, which, exclusively among the three proteins, serves as a hub in insulin signaling. PDB ID selected for IR is (PDB ID: 1IR3 [123]).

To identify possible active sites, CDD BLAST [124] and Metapocket server [125] were used. Later, the online server for the Lipinski filter was used for the assessment of the molecular properties for the retrieved ligands [126]. Filtered properties included the logP ratio (for the octanol : water partition coefficient), hydrogen bond donors/acceptor, and molar refractivity [126]. The computational prediction of absorption, distribution, metabolism, excretion, and toxicity properties was evaluated using the ADME-TOX drug v3.0 tool [127].

The molecular docking (MD) analysis played a key role in the screening process. Binding free energy filter and dissociation constant using YASARA [128] were used to predict the compatibility of six plausible bioactive compounds (herbacetin, kaempferol, gossypetin, leucodelphidin, sorbifolin, and leucoperalgonidin). Out of the six compounds, sorbifolin and herbacetin were found as the best suitable ligands for IR, AR, and SIRT-6 found in *Ficus lacor* Buch. and *Thespesia populnea*, respectively.

Additionally, the bioactive components present in *Pinus roxburghii* were screened against potential targets for diabetes via MD [129]. Target receptors included insulin receptor (IR), aldose reductase (AR), protein tyrosine phosphatase 1-beta (PTP-1*β*), and dipeptidyl peptidase-IV (DPP-IV). Here, we concentrate on the results obtained for protein tyrosine phosphatase 1-beta (PTP-1*β*) and the insulin receptor (IR). The 3D crystal structures of the receptors were obtained from Protein Data Bank (PDB). These included IR (PDB ID: 1IR3 [123]) and PTP1*β* (PDB ID: 2F70 [130]). Docking software used was the Molegro Virtual Docker (MVD) (Molegro ApS, Molegro Virtual Docker, vol. 2.4, ApS, Aarhus, Denmark, 2008.). The scores of docking experiments showed that cedeodarin, pinoresinol, and secoisoresinol had the remarkable docking results on the insulin receptor IR, PTP1*β*, and most remarkably the AR receptor. Next, LigandScout was used to build up the required pharmacophore model for active targets.

Moreover, pharmacophore models prepared using LigandScout predicted that His110 and Tyr48 in three receptors (AR, IR and PTP1*β*) are required to form the H-bond with the ligand. Specifically for IR, Asp1150, Met1079, and Leu1002 are suggested to be the most distinguished binding residues [120]. On the other hand, secoisoresinol, which has the highest MolDock score in the PTP1*β* analysis, showed interaction with His110. Besides, amino acid residues, namely, Ser1006, Lys1030, Asp1083, Met1079, and Glu1077, were the primary residues used in the interaction of the internal ligand and almost all of the active constituents in IR and PTP1*β* [129].

Next, extra ligands were predicted based on the pharmacophore model. ChemSpider database was used to retrieve the mol files and smile formulas of ligands. Marvin sketch was used to draw the structures of ligands, while energy minimization was performed using MMFF94 force field [131]. QSAR studies were then implemented to detect potential activators of biological objects. The calculation of important molecular properties and prediction of bioavailability were investigated via Molinspiration, an online tool [132]. In order to determine the protein-ligand interactions and ligands that passed the bioavailability test, IR was tested with molecule ANP (phosphoaminophosphonic acid-adenylate ester), and PTP1*β* (PDB ID: 2F70) was tested with UN608 (3-([3-(3-sulfoamino-phenyl)-propionylamino]-methyl)-phenyl)-sulfamic acid. Docking results showed positive interactions and good free energy results between the protein-ligand pairs studied in the pharmacophore model [129].

At a later stage, we investigated further interactions of one more protein of the tyrosine-protein phosphatase family, specifically PTP1B. One of the well-known interactions is with the 3-bromo-4,5-bis(2,3-dibromo-4,5-dihydroxybenzyl)-1,2-benzenediol (CYC31) bromophenol protein tyrosine phosphatase 1B (PTP1B) inhibitor isolated from the red algae *Rhodomela confervoides* [133]. Moreover, the influence of CYC31 on the insulin signaling was investigated via MD [109]. The crystal structure of PTP1B (PDB ID: 3QKP [134]) was acquired from the Protein Data Bank of RCSB, and later, AutoDock 4.0 program was used to perform the required MD [135]. The original ligand binds to the active site in the crystal structure [136, 137]. The ligand was removed and CYC31 was imported. The compound positioned itself in the catalytic site of PTP1B (PDB ID: 3QKP [134]) with a good docking score. A diphenol group formed a hydrogen bonding network with the N–H of residues Arg221 and Ala217. The middle phenyl ring formed three hydrogen bonds with residues Gln266 and Gly183 via its hydroxyl group.

### 5.2. PI3K, Akt, and mTOR

#### 5.2.1. Polyphenolic Compounds Target PI3K/Akt/mTOR Signaling

Myricetin, luteolin, quercetin, and morin are some of the naturally polyphenolic compounds found in fruits and vegetables. Such compounds are able to oxidize the C-ring of the basic structure of benzo-*γ*-pyrone to different ranges and differentiate between them. Similarly, emodin is anthraquinone laxative resin naturally found in the barks and roots of several plants, lichens, and molds [138]. This study further analyzed PI3K signaling proteins (phosphatidylinositol-4,5-bisphosphate 3-kinase), Akt (protein kinase B which is a serine/threonine-specific protein kinase), PDK1 (3-phosphoinositide-dependent protein kinase 1), and mTOR (serine/threonine-protein kinase mTOR) through a set of docking experiments. The required crystal structures were acquired from the Protein Data Bank as follows: Akt (PDB ID: 3MV5 [139]), PDK1 (PDB ID: 3RWQ [140]), PI3K (PDB ID: 3S2A [141]), and mTOR (PDB: 4DRI [142]).

The chosen ligand set consisted of 51 natural compounds along with 17 reference compounds selected from published literature. Maestro 9.3 (Schrödinger Inc.) [138] was used in the docking protocol between the ligands and protein molecules. The following ligands morin, quercetin, emodin, luteolin, and myricetin returned good docking score based on the free energy. Besides, throughout the docking process, the best binding confirmation results occurred in the analysis of PDK1 and PI3K.

The molecules were indeed further screened for their pharmacodynamics and pharmacokinetic characteristics, including compliance with the “Lipinski's Rule of Five” and the scores were trustworthy. Poor ADME properties remain an obstacle causing most of drug candidates not to pass the clinical trials. Using QikProp application [143], the ADME/T properties of compounds with best docking results were predicted [143, 144]. The achieved bioavailability presented promising results for quercetin, morin, luteolin, and emodin, while myricetin results were not as good. As a result, optimization of myricetin is needed to have better bioavailability.

As a positive control, sulforaphane administration is also known to impede pulmonary metastasis and progression of prostate cancer in TRAMP mice through suppressing the Akt signaling pathway [145, 146]. The docking score of sulforaphane against Akt presented promising results, but it showed less stable binding complex than the aforementioned natural compounds. The docking studies conducted put forth that sulforaphane had similar binding conformation and interaction profile to curcumin against Akt. Thus, it forms a plausible Akt inhibitor [138].

Morin, myricetin, and quercetin are different from each other considering the position of substitution or raw addition of the hydroxyl groups on the phenyl moiety. Both chromone as well as phenyl moieties were reported to be essential for protein-ligand interactions. Hydrogen bonds involved in protein-ligand interactions in both phenyl or chromone were found in the backbone and with a side chain. Considering the structure of PDK1 complexed with myricetin, hydrophobic interactions and hydrogen bonding were found to be crucial in stabilizing protein-ligand interactions. Amino acids included in these interactions are Leu159, Leu212, Ala109, Tyr161, Leu88, Ala162, Val143, and Ala162, being considered in hydrophobic interaction; and Thr222, Glu209, andSer160, being involved in hydrogen bonding and *π*–*π* stacking. The amino acids of PI3K such as Phe961, Trp812, Ile879, Ile 881, Val882, Tyr867, Ala885, Met 953, and Pro810 were key players in forming hydrophobic interactions and hydrogen bonding with quercetin, morin, and luteolin.

#### 5.2.2. Combinational Therapy and Computational Tools for Drug Scanning: PI3K/Akt/mTOR Pathway as the Target

Using different methods, an attempt was undertaken recently to design a combination therapy [147] of drugs inhibiting two or more proteins of the PI3K/Akt/mTOR pathway [148]. In this respect, available computational tools can help predicting many combinations against phosphatidylinositol-3-kinase (PI3K), protein kinase *b* (PKB/Akt), and mammalian target of rapamycin (mTOR). A virtual library of drugs was prepared using 1803 drugs approved by the FDA that were acquired from the approved drugs (Drug Bank Release Version 5.0.1) and appear to have optimal ADME/T parameters. To carry out MD studies, the crystal structures of the proteins Akt, PI3K, and mTOR (PDB ID: 3MV5 [139], 4JPS [149], and 3QAR [150]) were used. Protein processing, energy minimization, and docking simulations were carried out in the Protein Preparation Wizard in Maestro version 10.2, Schrödinger, LLC, New York, 2015, and the evaluation was based on GlideScore [151] for the binding interactions between the receptors of the pathway and the proposed drugs. All of the 1803 drugs were subjected to docking. The most favorable hits could bind mainly to the active site amino acids at the target proteins.

The natural ligands that were able to bind to effector proteins in the insulin signaling cascade include wortmannin, mitoxantrone, quinostatin, riboflavin, rapamycin, sirulimus, theophylline, 5′AMP, and ATP [148].

According to docking results, the interaction of vemurafenib was situated in the binding domain of Akt, with the following sets of interaction types: hydrogen bonding made with the amino acid residues Glu226 (N…N-H: 1.58 Å), Glu234 (N-H…O: 2.09 Å), and Ala230 (O-H…N: 2.35 Å); *π*-stacking with Tyr272 at a distance of 4.76 Å; and hydrophobic interactions with the following amino acids: Val164, Ala177, Ilu800, Thr291 and Met28. GlideScore of docking showed that riboflavin interacts nicely with the mTOR receptor. Moreover, two hydrogen bonding interactions were presented along with active site amino acid residues Lys890 at 2.07 Å and Val882 at 1.82 Å. Wotmannin, MK-2206, LY-294002, mitoxantrone, and rapamycin showed good interactions with at least two of the pathway proteins.

Based on the results of the MD method, running cost, and availability, the combination vemurafenib and riboflavin was further experimented in vitro, using the Western blot assay. Cells had a treatment phase with vemurafenib and riboflavin at 1 and 50 *μ*M concentrations, respectively, for 48 h time period. The experimental results showed interdependent effects supporting docking findings [148].

#### 5.2.3. Del, Combination of Docking with Experimentation: PI3K/Akt/mTOR as the Target

In silico MD analysis was combined with the experimental work (combined binding constant (Kd), kinome-level screen, and surface plasm on resonance (SPR)) to study the interaction affinity between Del (delphinidin [3,5,7,3′-, 4′-,5′-hexahydroxyflavylium]) and protein targets mTOR, PI3Ks (*α*, 2C*β*, and *γ*), p70S6K, and Akt [152]. Additionally, these enzymes are repressed in vitro in cultured normal human epidermal keratinocytes (NHEKs) when Del is added. Topical application of Del in the in vivo mouse model considerably relieved IMQ-induced psoriasis-like skin lesions in Balb/c mice [152]. Del is reported as a novel specific inhibitor of both serine/threonine (mTOR/p70S6K) and lipid (PI3Ks) kinases.

AutoDock4 analysis predicted strong binding of Del along with PI3K isoforms (PIK3CG, PIK3C2B, and PIK3CA, with binding energy of −8.69, −7.82, and −6.64 kcal/mol, respectively). Del orients in two binding sites (A and B) in PI3K*α* (alpha) kinase (PDB code 4JPS [149]). In site A (ATP phosphate binding site), Lys802, Asp810, and Asp933 bind to Del orient to form H-bonds to backbone atoms of Val851 at the nucleotide end. In site B (nucleotide binding domain), the ligand orients in reverse direction, and three hydroxyl hydrogens bind to the backbone of Val851 and Gln859 side chain. The binding free energy of cluster A is −6.63 kcal/mol and of cluster B is −6.64 kcal/mol.

As in PI3K*α*, Del binds in the two preferred sites for PI3K*γ* (gamma) kinase (PDB code 1E8X [153]). In position A, the hydroxyls of the phenyl ring bind to Lys833 in the lysine/aspartate-rich phosphate binding site, with the remaining hydroxyls binding near the nucleotide binding region. Hydrogen bonds therein interact with the amide and carboxyl of Val822 backbone. In position B, one of the three phenyl-OH binds to a backbone amide of Val882. A terminal hydroxyl interacts with the backbone amide of Asp844. Site A had the highest binding energy (−8.69 kcal/mol), and binding energy at site B showed an average of −8.45 kcal/mol. This complies with the chemistry being lower in the alpha isoform than in the gamma docking (a more stable binding in the gamma isoform).

Using tools in the Swiss-Model repository website [154], PI3K–C2*β* was homology fitted to the crystal structure of PI3K-*δ* (delta) (PDB code 4XE0) [155]. Over a stretch of around 1500 amino acids, a 32.09% sequence similarity was found. While the binding site is highly similar from a structural perspective to the PI3K*γ* binding site, it has several distinct amino acids. As a result, AutoDock4 protein-ligand (PI3K–C2*β*-Del) docking predicted that Del only binds at an energy of −7.82 kcal/mol with a very different cluster to the gamma isoform. On the other hand, reference NVP ligand binds at an energy of −9.34 kcal/mol. PI3K–C2*β* was shown to have a single backbone carbonyl involved in the binding of Val1115, while the reference molecule had multiple hydrogen bonds.

Del showed no binding to Akt in the binding assay. Thus, two crystal structures (PDB codes 1UNQ and 3D0E) [156, 157] were utilized to study plausible interactions not covered by AutoDock. In both 1UNQ and 3D0E, lower affinity of Del-Akt binding was found when compared to the reference structure with a known inhibitor, with binding free energies of −6.15 (1UNQ) and −7.73 (3D0E) kcal/mol, respectively.

On the other hand, ribosomal protein S6 kinase beta-1 (S6K1), referred to usually as p70S6 kinase (P70S6K), is a downstream target of the PI3K/Akt/mTOR pathway, which was reported to bind to Del. This binding was further investigated by docking Del in the P70 kinase site in P70S6K1 (PDB code 3A60) [158]. Binding was found to be in a single cluster (binding free energy = −6.97 kcal/mol). It included the three hydroxyl groups of Del to bind in kinase site, which were found to bind to the backbone in the nucleotide binding site. At the other end, two hydroxyls could bridge Glu143 and Lys123 in the phosphate end of the Akt binding pocket.

The interaction between Del and mTOR was based on the 4JSP crystal structure (PDB code 4JSP) [149]. Two binding positions existed that have approximately equal binding free energies (A with −7.91 kcal/mol and B with −7.33 kcal/mol) in two clusters. In site A, Del binds to the Lys-Asp pair to chelate phosphate in ATP. In site B, Del binds many hydrogen bonds in the backbone nucleotide loop [152].

#### 5.2.4. Naphthoquinone Analogs: PI3K/Akt/mTOR as the Target

On the other hand, structural computational biology approaches were applied on a diverse group of naphthoquinone analogs that can inhibit crucial proteins involved in cancer signaling to find plausible effective anticancer drugs [159]. These compounds were used in the screening against the three target proteins (PI3K, Akt, and mTOR). The naphthoquinones are a class of organic compounds derived from naphthalene and include a broad set of plant metabolites [160]. The naphthoquinone analogs include many natural products, e.g., juglone, lapachol, menatetrenone plumbagin, lawsone, alkanin, and many synthetic compounds including atovaquone and menadione [160]. The naphthoquinone analogs were retrieved from PubChem [161] as compounds similar to 1,4-naphthoquinone. Later, filtering based on the “Lipinski's Rule of Five” [126] resulted in 954 compounds.

The 3D structures of human mTOR, PI3K p110*γ* (PI3K*γ*), and Akt1 were obtained from the Protein Data Bank (PDB) with PDB IDs 3L54 [162], 3MVH [139], and 4JT6 [163], respectively. The selected proteins included a complete kinase domain with an ATP-binding site. MD and dynamic simulations have been successfully used to identify drug candidates [164–166]. Dock v.6.5 was used to complete the virtual screening of the naphthoquinone analogs against mTOR, Akt1, and PI3K*γ* [167]. The X-Score v.1.2.11 was applied to evaluate the binding energies and dissociation constants [168]. Additionally, the loss in the accessible surface area (ASA) resulting from ligand binding was calculated in order to measure the degree of involvement of residue in binding. The “pkCSM-pharmacokinetics” online web server was used to predict drug likeness and pharmacokinetic ADME/Tox properties [169]. A training set of compounds was used to predict pharmacokinetic properties of plausible drugs.

The overall analysis identified the common compound found among the top 10 dock score lists of mTOR, Akt1, and PI3K*γ*, which is the one labelled with the PubChem Compound ID, CID: 20759629. Therefore, such a compound was proposed as a promising inhibitor for 3 protein kinases. The compound has the molecular formula “C_22_H_14_O_2_,” and its IUPAC name is “(4Z)-4-[(2E)-2-(4-oxonaphthalen-1-ylidene)ethylidene]naphthalen-1-one.”

Moreover, the MD study pointed that the CID-20759629 bound in the ATP-binding site lines the residues Met804, Ser806, Trp812, Ile831, Lys833, Tyr867, Ile879, Asp950, Asn951, Met953, Ile963, and Asp964 in PI3K*γ*. CID-20759629 docked in the ATP-binding site in Akt lines the interacting residues Phe442, Gly157, Leu156, Ala177, Met227, Val164, Glu228, Ala230, Thr211, Glu234, Thr291, Asp292, Met281, and Phe438. Moreover, the MD study showed that CID-20759629 is binding in the active site in mTOR using the following residues: Asp2357, Leu2185, Asp2195, Glu2190, Tyr2225, Gly2238, Ile2237, Trp2239, Met2345, Val2240, and Ile2356. LigAlign v.1.00 was used to compare the binding of CID-20759629 in the active sites of mTOR, Akt1, and PI3K*γ* [170]. Many of the listed above residues of the three proteins were found to fall in close proximity in the binding site of the three proteins and were called equivalent residues.

#### 5.2.5. The Flavonoid Hesperetin and Akt as Targets

Flavonoids were recently targeted by MD studies [171]. MD was conducted to study the inhibitory nature of the hesperetin molecule [172] referred to frequently as 3ʹ,5,7-trihydroxy-4-methoxy flavanone (C_16_H_14_O_6_), which is one of the flavonoids, specifically from the flavanone subclass [173].

The optimization process of the lead compound was performed using B3LYP (Becke 3-Lee-Yang and Parr) level of theories [174, 175] in Gaussian 09 program [176] and many MD studies accomplished in AutoDock (1.4.6 version) software [177] for hesperetin-Akt1 combination (PDB ID for Akt1: 3O96) [178]. To view the protein-ligand complex along with the intermolecular interactions between the protein and ligand molecules, PyMOL [179], Chimera [180] and Discovery studio programs were incorporated. In the docking experiment, the selected protein functional sites were evaluated while calculating the minimum binding energy. The minimum binding energy of −6.10 kcal/mol and intermolecular energy of −7.60 kcal/mol were reported from interaction. Exactly three residues could form hydrogen bonds with hesperetin: Leu210, Gln79, and Thr211 with bond distances of 2.4, 2.2, and 2.0 Å, respectively. The study was combined with an in vivo study that showed an antiproliferative effect of hesperetin towards human lung cancer cells [171].

#### 5.2.6. The Flavonol Herbacetin with Akt as a Target

Oridonin from *Rabdosia rubescens* is an ATP competitive inhibitor of Akt1 and Akt2 isoforms [181, 182]. Shogaol, extracted from ginger root, does not inhibit PI3K or mTOR, while it does inhibit Akt1 and Akt2 [183]. In silico models have shown that the flavonol herbacetin, present in flaxseed and ramose scouring rush herb, acts as an inhibitor on Akt1 and Akt2 through forming hydrogen bonds into ATP-binding pocket [184].

#### 5.2.7. Natural Product Repository for Akt, Q7G as the Best Hit: Structure and Ligand-Based Approaches

We further investigated one additional serine/threonine-specific protein kinase which is RAC-alpha serine/threonine-protein kinase (Akt1). Recently, the natural product repository for Akt1 was screened using in silico structure and ligand-based methods [185]. In this study, structure-based and ligand-based strategies were used to evaluate Akt1 possible inhibition including 700 pure natural products from which the test compounds are acquired. Among the tested compounds, only 8 newly discovered had trustworthy results showing inhibition at 500 nM through in vitro screening. The eight new compounds are ovalitenone (IN00551), 6-0-feruloyl catalpol (IN00145), isobutrin (IN00262), MBH-4-flouro racemic (IN00453), irigenin (IN00498), 5,7-dihydroxy-8-(3-methyl-2-butenyl) coumarin (IN00238), junipeginin C (IN00500), and 5,7,4′-trihydroxyflavone-3-O-glucuronide (IN00510). All of them are phytochemicals in plants such as *Arnebia euchroma*, *Butea monosperma*, *Capsicum annuum*, *Colebrookea oppositifolia*, *Dysoxylum binectariferum*, *Euphorbia hirta*, *Iris hookeriana*, *Picrorhiza kurroa*, *Juniperus macropoda*, *Ophiorrhiza mungos*, *Toddalia asiatica*, and *Scrophularia dentata*. Some plants, e.g., *Euphorbia hirta* and *Picrorhiza kurroa* have been reported to have anticarcinogenic properties [186]. The authors of this study performed MD studies of Akt1 with only the most active compound IN00145. The final results presented that Asp292, Phe161, Glu191, Lys179, Ala230, Glu228, Glu278, Thr291, and Glu234 residues are essential for their role in stabilizing the formation of the protein-ligand complex of Akt1 and IN00145.

The following PDB codes were considered for 11 Akt structures: 4GV1, 3CQW, 3CQU, 3MVH, 3MV5, 3OW4, 3OCB, 3QKL, 3QKM, 3QKK, and 4EKL [139, 187–193]. The protein preparation tool of Schrodinger software was used to depict the ligand binding sites based on the already cocrystallized ligand using Glide [151, 194]. On the other hand, 35 possible Akt1 ATP competitive inhibitors were grouped from the literature. Similarity search was conducted for the inhibitors with exactly 11 cocrystallized inhibitors of Akt1. Additionally, to build ligand-based pharmacophore models, five congeneric series, categorized into set-A to set-E, were used. Set-A was the 2,3,5-trisubstituted pyridines [195, 196], set-B included 5-pyrrolopyridinyl-2-thiophenecarboxamides [197], set-C fits in pyrrolopyrimidine [187], set-D encompassed dihydrothieno and dihydrofuropyrimidines [189], and set-E included isoquinoline–pyridine with derivatives of oxindole–pyridine [198, 199]. 3D-QSAR models were prepared on all of the sets to complete the validation process. The test results confirmed that the compounds in all sets support the similar features of the known Akt1target inhibitors [185]. With these findings, the authors confirmed that the hydrophobic residues comprising the Akt1 final binding site are Ala177, Tyr229, Met227, Phe237, Ala230, Phe236, Tyr437, Met281, Phe442, Leu156, Phe438, Val164; polar Asp279, Thr211, Thr435, Thr291, and Thr443 residues; negatively charged Glu234, Asp439, Glu278, Asp292, and Glu228 residues; and positively charged Arg241, Lys179, and Lys158.

Furthermore, a pharmacophore model was prepared to identify potential possible Akt1 allosteric inhibitors among a library of natural compounds [200]. As a reference structure, the crystal structure of Akt1 along with the inhibitor VIII (PDB ID: 3O96: [178]) was acquired from the RCSB Protein Data Bank (PDB) [201]. The final results were filtered by docking onto the Akt1 allosteric site considering the default VSW (virtual screening workflow) parameters.

Based on the interaction profile with the allosteric residues, including hydrogen bonding, polar contacts, and salt bridges, quercetin-7-O-*β*-d-glucopyranoside (Q7G) was reported as the best among all selected molecules. The interactions with Q7G were found to help in stabilizing Akt1 in the inactive conformation with a minimum binding free energy throughout the MD simulation.

At the experimental level, Q7G initiated a dose-dependent breast cancer cells (MDA MB-231) inhibition, pressuring them to remain in the G1 phase. Concomitant downregulation of Bcl-2 and upregulation of cleaved caspase-3 and PARP were also noticed. A confirmed interaction between Akt1 and Q7G was also shown (with a dissociation constant (K_d_) of 0.246 *μ*M) [200].

#### 5.2.8. Inverse Virtual Screening with Akt and PTEN as Targets

One way to study how natural products would affect target proteins is the scan of a large number of biological targets to evaluate their interaction with the target ligand. Experimentally, an obstacle lies at the pharmacological level in the limited availability of ligands and low production levels by organisms to screen a large number of proteins. The process of many pharmacological tests against many receptors is frequently prohibited because of some compounds that are obtained from natural sources. To reduce such an effect, a new computational tool, the inverse virtual screening, was used to facilitate new drug discovery [202]. Inverse virtual screening and MD were used to analyze a database that contains 43 small natural molecules that were formerly tested for their possible activity (antiangiogenic, antitumor, antiproliferative, cytotoxic, and activity on the cytoskeleton), also tested against a group of 126 receptor sites involved in different forms of cancer [203]. Akt and PTEN were among the tested target proteins. To perform the final calculations, AutoDock Vina was used [177].

The following natural hits were found to have the best docking scores and were docked against several targets: 6-methylheptyl sulfate [204] and aegelinol [205–207] with 18 targets, iodocionin [208] with 17 targets, 2-hydroxynephthenol [209] with eight targets, (Z)-oct-5-enyl sulfate [204] with 17 targets, and osthol [210] with 14 targets. Moreover, these compounds targeted normally Akt, ABL2, CDK6, BAB1, mTOR, cathepsin B, PYK2, cathepsin K, and EGFR.

#### 5.2.9. Flavonoids and Virtual Screening to the ATP-Binding Site of PI3K*γ*

Another project analyzed the ability of flavonoids and compounds similar to flavone to inhibit PI3K*γ* [211]. Virtual screening and MD to the ATP-binding site of PI3K*γ* were applied on 1173 selected compounds. Considering the docking score, only the highest 10 compounds in score were labelled as potential PI3K*γ* inhibitors and intensively evaluated through binding analyses. Indeed, their binding affinity results were similar to native PI3K*γ* inhibitors. Finally, the authors presented the PubChem CID of the 10 compounds ranked in a descending order: “53463223” (ACMC-20dh6x; 2H-1-benzopyran-3-ol,2-[3,4-bis(2-hydroxyethoxy)phenyl]-3,4-dihydro-5,7-bis(2-hydroxyethoxy)-(2R-trans)-(9CI)), “71260095” (SCHEMBL14700159), “131834212” ([2-hydroxy-3-(methoxymethyl)-6-(3,5,7-trihydroxy-3,4-dihydro-2H-1-benzopyran-2-yl)phenyl]oxidanesulfonic acid), “21676336” ((2R,3S,4S)-2-(3,4-dihydroxyphenyl)-3,5,7-trihydroxy-3,4-dihydro-2H-chromene-4-sulfonate), “45277410” (CHEMBL1088329), “44326949” (CHEMBL327396), “123270861” (tert-butyl [2-(3,4-dihydroxyphenyl)-5,7-dihydroxy-3,4-dihydro-2H-chromen-3-yl]oxymethyl carbonate), “156200” (2H-1-benzopyran-5,7-diol, 3-(3-aminopropoxy)-2-(3,4-dihydroxyphenyl)-3,4-dihydro-, (2R-trans), “71634-93-0” (DTXSID30221882), “131801248” (CHEBI:138950), and “‘132256977” (SCHEMBL19743905) [211].

#### 5.2.10. Virtual Screening of a Gigantic Library of Compounds with PI3k*α* as a Target

In a thorough study, structure-based drug design protocol was used to uncover novel classes of PI3K*α* inhibitors among 240,000 compounds [212]. Virtual screening, docking simulations, and in vitro enzyme assay were performed. A solvation model was applied in calculating the binding free energy between PI3K*α* and the suggested ligands, which could help to positively find good leads for enzyme assay [213].

The 3D structure of PI3K*α* (human p110 alpha/p85 alpha) in the resting form (PDB code: 2RD0) [214] was selected for virtual screening and docking simulations. The library used in the docking of PI3K*α* included about 240 thousand compounds, retrieved from the chemical database distributed by InterBioScreen (http://www.ibscreen.com). Prior to docking, molecules were filtrated on the basis of Lipinski's “Rule of Five” to determine ligands that obey the rule [126]. Suitable AMBER force field [215] parameters were chosen to aid in calculation of the van der Waals interactions and the internal energy of a ligand as originally implemented in the AutoDock program [216]. The simulations performed using the AutoDock program were all applied exactly in the defined PI3K*α* ATP-binding site in order to determine and rank the compounds in the docking library based on the binding affinities calculated.

Of the 240 thousand compounds involved in virtual screening during docking simulations, only the top 100 compounds according to the score were considered later as virtual hits. The selected compounds were tested in high-throughput binding assay (KINOMEscan, Ambit Biosciences) over PI3K*α* at a concentration of 10 *μ*M [217]. Percent of control (POC) values for four compounds (compound 1: (E)-2-amino-N-(amino((4-methylquinazolin-2-yl)amino)methylene)benzamide; compound 2: N-(6-methylpyridin-2-yl)-5-((4-oxoquinazolin-3(4H)-yl)methylfuran-2-carboxamide; compound 3: (E)-2-ethoxy-6((furan-2-ylmethylene)amino)acridin-9-amine; and compound 4: N-(4-(5-furan-2-yl)-1,3,4-oxadiazol-2-yl)phenyl-2-(p-tolyloxy)acetamide) were found to be less than 70 and were considered for the determination of the values of IC50. IC50 values for the four inhibitors ranged from 20 to 40 *μ*M, what suggests moderate inhibition against PI3K*α*. Compounds 1 and 2 possess quinazoline and quinazolin-4-one groups, respectively, what mimics the ATP adenine group.

The binding modes of the four compounds in the PI3K*α* ATP-binding site were comparatively analyzed, where the docked structures were superposed. The hydrogen bond donors in the inhibitors were found to point toward the backbone groups at the PI3K*α* gatekeeper site. The hydrophobic groups were found to be located between the PI3K*α* two loop structures at the top of C-terminal domain. Moreover, no binding configurations were reported regarding the inhibitor residing outside the defined ATP-binding site. This supports the possibility that the inhibitors target the catalytic ATP-binding site of PI3K*α*, what rules out allosteric inhibition [212].

#### 5.2.11. Pharmacophore Modeling Based on Known PI3K Inhibitors

Along the same side, new potent PI3K inhibitors [218] were identified using a ligand-based pharmacophore model combined with MD studies [219]. Phase module was used to generate the pharmacophore models [220], based on the known series of 46 PI3K*α* inhibitors selected from literature [221]. These were classified as 2-aminothiazole (S)-proline-amide-urea series, with their biological activities (IC_50_) ranging between 1.8 and 10004 nM. The given IC_50_ values were changed to the pIC_50_ scale (−logIC_50_) to be more easily utilized in the generation of pharmacophore modeling [219]. The Guner–Henry scoring method was used for the generation and validation of many pharmacophores. The best models were used to query ligands (synthetic and natural) from the ZINC database [222]. Further validation for the retrieved hits was also carried out using ADME properties and Lipinski's Rule of Five [126]. Four compounds were determined using this protocol, what introduces them as potential novel lead compounds for designing PI3K inhibitors.

MD was carried out for ligand-PI3K complexes using SurflexDock interfaced with SYBYLX 2.0 (SYBYL Software, version 7.3, Tripos Associates Inc., St. Louis, USA, 2006, http://www.tripos.com.), which also compares the interaction of known inhibitors to the defined hits. Energy minimization was carried out for 100 steps.

The binding site of PI3K was found to include the following residues: Phe294, Phe87, Phe88, His39, Phe111, Aan114, Met115, Phe112, Tyr166, Arg170, Phe116, Asp185, Tyr184, Leu187, Val186, Leu206, Lys210, Gln203, Trp259, Phe211, Tyr262, Gly260, Phe265, Ser266, His263, Glu269, Leu267, Trp283, Leu286, Ala270, Leu292, Phe288, Val289, Pro287, Thr290, Ser291, and Ala293.

#### 5.2.12. Pharmacophore Modeling Based on IQO Inhibitor with Akt as the Target

Moreover, a recent study conducted computational screening for natural “lead” compounds that can plausibly bind Akt protein and modulate its activity [223]. A pharmacophore model was generated based on the experimental structure of Akt1 in complex with IQO, an inhibitor. Ser205 was found to participate in H-bond interaction with IQO. Other types of interactions, including hydrophobic interactions, were formed by a number of other amino acids. The minimal pharmacophore model had four features: one aromatic ring, two hydrophobic regions, and one hydrogen bond acceptor. To match the pharmacophore model, screening for natural compounds was performed using the ZINC database [222]. This generated several candidates. The best ones were further screened using MD and ADME/Tox analyses.

Docking results have shown that known inhibitors of Akt1 interact with a low binding energy (<−12 kcal/mol). The binding energy for the selected molecules had values around −10 kcal/mol, which is good enough to initiate binding. There was a good match between the docking results and experimental work regarding the negative control, quercetin, with a high binding energy of approximately −6 kcal/mol. The results of ADMET analysis suggest that ligands can be absorbed by the intestinal barrier, but cannot pass the blood-brain barrier. The toxicity profiles of the selected compounds, analyzed via TOPKAT software integrated into Discovery Studio, reported no mutagenic or carcinogenic effects. The selected molecules, ZINC2429155 (STL1), ZINC1447881 (AC1), and ZINC02161363, were fished as predicted lead compounds. Nonetheless, ZINC02161363 has not been experimentally tested due to solubility issues.

Thus, two compounds were further subjected to experimental validation: ZINC2429155 (STL1) and ZINC1447881 (AC1). IQO was used as the positive control, while quercetin was used as the negative control. Quercetin is experimentally suggested as not being a direct ligand of Akt1 [224, 225]. Of the two screened molecules, only STL1 inhibited Akt [223].

#### 5.2.13. High Throughput Screening for PI3K Inhibitors

Furthermore, high-throughput screening (HTS) was recently used to find natural or synthetic compounds that target PI3K with H1047 R mutation [226]. Using a combination of HTS and MD [227–229], new scaffolds of PI3K inhibitors, selective for either H1047 R mutant or the wild-type PI3K*α*, were screened. HTS experiments were performed using SYBYLX2.1.1. A set of statistical analysis tests were used to find whether the analyzed sets via HTS were selective for the H1047R mutant. MD was also performed via GOLD v5.2.2 software [230]. The human PI3K*α* structure (PDB: 2RD0 [214]) was used in the docking experiment [231]. A total of 288 thousand natural and synthetic compounds were screened, among which only 124 initial hits with considering the predicted binding mode were further selected. An 18 Å cavity centred on the CD1 atom of Ile800 was found to be the docking site. ChemScore was used to perform docking with a modified scoring function used specifically with kinases [232]. All known PI3K inhibitors form hydrogen bond interactions with the backbone amide of Val851 at the linker. Selective inhibitors of PI3K*α* bind to p110*α*-specific Gln859 amino acid [149, 233]. MD showed that of the 188 initial screened compounds, 157 could form with the Val851 linker a set of hydrogen bond interactions. However, exactly three hits were reported to form a set of hydrogen bonds with the polar groups of Gln859 (WNN0429-D004, WNN1560-A006, and WNN4101-D008), as in the PI3K inhibitors formerly discussed [149, 233–235]. Other two hits (WNN1237–B004 and WNN1489–B003) have a hydrogen bond donor–acceptor motif similar to kinase inhibitors [163, 236].

Based on the docking results, twenty-four compounds were furthermore analyzed for concentration-dependent responses via calculating IC_50_ values against both H1047 R mutant and wild-type enzymes. It was found by probing the in vitro catalytic activity that most of the confirmed hits were not selective for H1047 R PI3K*α* over wild-type PI3K*α*, which presents that it is insufficient to consider one single mutation to achieve selectivity. This matches well with previous studies that depicted the elevation in activity of the H1047 R mutant to be due to the elevated membrane binding but not catalytic site activity [237–239].

#### 5.2.14. PDK1 as a Target for 7-Azaindole

The 3-phosphoinositide-dependent kinase 1 (PDK1) is a key regulator of the oncogenic PI3K signaling pathway [240]. To investigate this area further, a hit-to-lead chemical optimization was carried out for the 7-azaindole series, which are hinge binders for protein kinases [241]. The identified 7-azaindoles were found to be structurally related to the known natural products meridianin A, variolin B, and also the synthetic analog meriolin 1, where already micromolar PDK1 inhibition (IC_50_ = 5.4 *μ*M- 5.9 *μ*M) was reported [242–244].

The 7-azaindoles reported with low micromolar IC_50_ (e.g., 16: IC_50_ = 1.1 *μ*M) were used in the biochemical PDK1 assay. 7-Azaindoles had considerably potent biochemical PDK1 activity in the nanomolar range. Nonetheless, analogs only showed moderate activities (42: IC_50_ = 2.3 *μ*M). The PDK1 X-ray structures along with early ADME analysis plus the structure–activity relationship all together could provide the basis to optimize the subsequent hit-to-lead. Using HTS, focused kinase library, and virtual screening, the inhibitors 4-(1H-pyrrolo[2,3-b]pyridin-3-yl)pyrimidin-2-amine and 4-butyl-6-(1H-pyrrolo[2,3-b]pyridin-3-yl)pyrimidin-2-amine, comprising a 7-azaindole ring as two-contact hinge binder, were among the most potent hits. Both 7-azaindoles showed moderate kinase selectivity.

#### 5.2.15. Sesquiterpenoids and Target Prediction

Sesquiterpenoids were found to have stereochemical minutiae, notable architectural diversity, and distinct biological activities [245]. Carainterol A is a natural product of sesquiterpenoids which was isolated from the aerial part of a known herb in traditional Chinese medicine that is *Caragana intermedia* [246]. A recent study used PharmMapper to predict target proteins that can plausibly bind to carainterol A and ClueGO to detect all related pathways [110]. A concomitant experimental procedure was applied, where carainterol A was tested specifically in HepG2 cells to elucidate its biochemical action. 118 predicted potential molecular targets were retrieved that have a diversity of pathological and/or physiological processes. Among them, 46 targets (39%) including RARG, INSR, MDM2, RXRA, PPARG, RXRB, PTN1, and IGF1R are related to diabetes. Of the ligand-protein interactions, sixteen interactions were found within a threshold for the fitting score of docking (greater than 3.5). Of those proteins, protein tyrosine phosphatase 1B (PTN1) works as a negative regulator in insulin signaling [247]. Agonists of retinoid *X* receptor (RXR) are insulin sensitizers in obese mice [248]. Another protein is the proliferator-activated receptor *γ* (PPAR*γ*). Mutations in this protein cause insulin resistance and the consequent type 2 diabetes [249]. Therefore, the interaction of carainterol A to these proteins seems to be important in glucose regulation and diabetes management.

Mapping of the target proteins to diabetes was handled using the TTD, PharmGKB, CTD, and KEGG databases. ClueGo analysis for multitargets was also performed. Molecular functions related to diabetes included insulin receptor binding, insulin receptor substrate binding, protein tyrosine kinase activity, steroid hormone receptor activity, and retinoid *X* receptor binding. The following pathways related to insulin signaling were included, among others, in the ClueGo results: the PI3K-Akt signaling pathway, the AMPK signaling pathway, and the insulin signaling pathway. Carainterol A showed no direct effect on GLUT4 translocation in experiment. Still, an unknown downstream signaling component of IRS-1 signaling might be the target [110]. As a result, carainterol A can function as a beneficial chemical targeting the insulin signaling pathway, and docking experiments to proteins in insulin signaling might show promising results.

### 5.3. Further Proteins Effecting Insulin Signaling

On the other hand, we investigated a set of proteins that might have a direct effect on the insulin signaling. Initially, we started with the Ras-related C3 botulinum toxin substrate 1 (RAC1) signaling *G* protein. Rac1, a Rho-family GTPase, was found to be an intrinsic effector of GLUT4 translocation. Mobilizing GLUT4 to the cell surface, initiated through the activation of class I phosphatidylinositol-3-kinase (PI3K) by insulin receptor substrate (IRS)-1, is a result of signal bifurcation. At the one hand, Akt2 activation and the consequent inhibition of its substrate AS160 lead to the activation of its target Rab GTPases, Rab8A, and Rab13 located in skeletal muscles. At the other end, Rac1 induces the remodeling of cortical actin filament through the Arp2/3 complex and cofilin [250]. RAC1 has been intensively targeted in the last decades due to its confirmed extraordinary regulation roles. For example, a chemobioinformatics workflow is conducted to address drug design aspects with special reference to RAC1 [251]. The pipeline included the chemical abstract system (CAS) data mining, structural bioinformatics analysis, docking, and exploratory statistics. The preliminary set of ligands was selected using CAS (Scifinder Panorama) [252]. The query used for data mining was RAC1. To this aim, The ZINC database was used. NSC23766 ligand, extracted from the ZINC database [222], was used as a reference ligand. As a result, 1988 ligand structures were obtained and classified into three major classes of chemical compounds: morpholines (528 hits), flavonoids (751 hits), and imidazoles (709 hits). It should be noted that recent studies have emphasized the inhibitory actions of flavonoids on Rho GTPase activity. No experimental evidence has yet suggested the confirmed interaction between RAC1 and flavonoids [253].

The PDB entry for the docking analysis of the protein target of RAC1 is 2FJU [254]. The structure shows several domains, including a core beta, an R-alpha larger core, and an L-alpha smaller core. Dock 6.2 [167] was employed in the calculations needed for the scoring scheme (virtual screening) [255] and to estimate binding energy for each ligand-protein complex. The strength of protein-ligand interaction has been evaluated by using the Kuntz's “ligand efficiency” (LE) parameter [256, 257].

It was previously noted that the reference compound NSC23766 interacts with RAC1 GTP binding domain [222]. Indeed, the three classes of ligands show a favored interaction with RAC1 GTP-binding domains. To define protein domains that surround the residues, Doolittle hydrophobicity profiles have been analyzed [258]. This profile also assesses solvent accessibility, which could show favorable ligand binding sites [251]. Only a subset of flavonoids (24 out of 751), morpholines (10 out 528), and imidazoles (15 out of 709) was found to preferentially bind with RAC1 exactly in the region: Asp65-Arg-66-Arg68-Pro69 and Ala95-Lys96-Pro99-Glu100-His103-His104.

Moreover, a recent study that used an inverse docking study was performed to assess the probability of rhein to be a sensitization agent with multitarget radiotherapy [259]. Rhein (1,8-dihydroxy-3-carboxy anthraquinone) is a natural anthraquinone compound that can be extracted from numerous Asian herbal medicines, such as *Polygonum multiflorum* and rhubarb [260]. The reports of the pharmacological activities of rhein include mainly the antitumor effect of rhein on cancer cells [260], e.g., in breast cancer, granulocyte leukemia, liver cancer, lung adenocarcinoma, stomach cancer, and tongue cancer [261–265]. The receptors that were tested for rhein binding included EGFR (3LZB, [266]), RAC1 (1G4U, [267]), CDH1 (3FF8, [268]), COPS2 (4D18, [269]), and HSP90 (5FNC, [270]), respectively.

To prepare the ligand, ChemBioDraw Ultra was used to draw, write, and save the dimensional structure of rhein as a PDB format [271]. Docking experiments were carried out using the docking algorithms employed in MOE [272]. Site Finder module of MOE was used to dig for the active site within the macromolecule. Hydrophobic pockets were considered as the active sites [272]. The Triangle Matcher method was used to evaluate the docking of ligand molecules within the active sites the receptors [273].

In this process, the insulin signaling-related proteins EGFR, CDH1, HSP90, and RAC1 are the suggested protein targets. Thirty docking conformations were ranked based on free energy values. Accordingly, the binding affinities of the five candidate proteins to rhein were ranked in descending order as follows: RAC1 > HSP90 > EGFR > CDH1 > COPS2. We concentrate in this review on RAC1 as a target protein in insulin signaling. RAC1 was found to form four hydrogen bonds with rhein, where rhein acted as a backbone H-bond donor and one hydrogen bond with rhein as a side chain donor. Rhein docked directly in the RAC1 binding cavity. Most amino acid residues contributed stronger hydrophobic van der Waals force interactions.

Similarly, a derivative of rhein, 4F, was investigated for its in vitro deregulation effects on RAC1 in breast cancer cells [274]. 4F was reported to have roles in cell migration and invasion and cytoskeletal change [274]. Several derivatives were synthesized from modifications in rhein structure with antitumor effects. Examples include derivative RP-4 [275] and derivative 4A [276, 277]. Derivative 4F showed especially good bioavailability. Indeed, it seems to have stronger inhibition than rhein on cell proliferation, migration, and invasion of breast cancer. It also causes cytoskeletal changes, represses RAC1 promoter activity in cells, and downregulates the RAC1 protein expression [274]. Molecular Operating Environment (MOE. 2008, CCG Montreal, Canada) [278] was used to dock several ligands intro RAC1 protein. The ligands included rhein, derivative 4F, and the positive control NSC23766. The conformational strength for binding of each ligand bound to RAC1 was evaluated. The binding stability of the three compounds to RAC1 is ranked in descending format from the strongest to weakest as derivative 4F > NSC23766 > rhein [274].

### 5.4. PTEN

Another part of our investigation targeted the phosphatase and tensin homolog (PTEN) protein which is a negative regulator of the PI3K/Akt/mTOR pathways. In a newly published in silico investigation, structure-based virtual screening, MD, molecular mechanics/generalized born surface area (MM/GBSA), molecular dynamics simulations, and prediction of ADME were conducted to find the drug likeness properties in addition to the binding affinity and stability of naringin and other compounds upon binding to PTEN [279]. Naringin (4′,5,7-trihydroxyflavanone 7-rhamnoglucoside) is a bioactive flavonoid, which naturally occurs in citrus fruits [280]. Due to the existence of 8 hydroxyl substituents (OH) in the naringin ring structure, it possesses antioxidant and anticancer activities, e.g., in human bladder cancer cells, breast cancer cells, and cervical cancer cells [281–283]. The antioxidant activity functions to upregulate the gene expression of superoxide dismutase, catalase, and glutathione peroxidase [284, 285]. Through increasing the expression levels for the tumor suppressor PTEN protein, naringin can induce an inhibitory effect on the signaling pathway (PI3K/Akt) in cancer cells [286]. Naringin-PTEN complex has a good binding stability, with no violations to Lipinski' Rule of Five. Naringin compound was found to have 89% human oral absorption, and the pharmacokinetic properties were reported as favorable, what predicts good bioavailability for the drug. However, while other screened compounds had good binding profiles, they violated the drug-likeness properties.

The following protocol was followed to conclude the results: the crystal structure of PTEN protein (PDB ID: 1D5R, [287]) was retrieved. After that, the protein preparation wizard, Schrodinger, LLC, NY, was used to preprocess the protein. The protein crystal structure minimization and optimization were performed using OPLS-2005 force field [288]. Naringin was retrieved from the PubChem database [161]. LigPrep module was used to assign possible ionization states at pH 7 in addition to bond angle and bond length [273]. OPLS-2005 force field was then used to energy-minimize the structure [289–291]. Virtual screening (VS) was completed with the help of Glide module (Schrodinger, LLC, NY, 2017) to select the best docking pose of the screened hits [292, 293], followed by docking experiments. Using a Glide-XP protocol, naringin was docked with the PTEN protein binding site [294], and the naringin-PTEN complex binding free energy was calculated [295, 296]. GROMACS package was used to run molecular dynamics simulation on the protein-ligand complex (50 ns run) [297]. To study the drug likeness properties of naringin and to find the pharmaceutical relevant parameters and also the pharmacokinetics properties, the ADME properties were calculated with the help of the Schrodinger QikProp module. Furthermore, Lipinski's Rule of Five was used to assess the drug properties [298].

The formation of hydrogen bond was found to significantly influence the interaction between naringin and PTEN protein. The following set of amino acids was previously reported to affect the functionality of PTEN: Lys 128, Arg130, Cys124, Gly127, Asp92, His93, Ala126, Gln171, and Lys125 [287]. Naringin was found to bind to some of these amino acids using docking studies. The following types of PTEN-naringin interactions existed: hydrogen bond, ᴫ-ᴫ stacking, ᴫ-anion interactions, and salt bridges. Residues involved were Lys330, Asp24, Arg47, Gly44, Tyr16, Asp92, His93, Val45, Lys128, Lys164, Ala126, Asp326, and Asp162. Naringin formed four hydrogen bonds with Val45, Ala126, Asp92, and Tyr16 residues [299]. Ala126, Val45, and Tyr16 are moderately hydrophobic in nature, what introduces stable protein-ligand complexes during the molecular dynamic simulation. Furthermore, Asp92-naringin binding was stabilized by strong interaction with the hydroxyl group of naringin molecule. Asp92 is a vital residue of PTEN catalytic pocket [300].

### 5.5. GLUT4

Our last protein of interest in this work is the glucose transporter-4-protein (GLUT4). Glucose is utilized by cancer cells at a higher rate than normal cells for their proliferation. Therefore, phytochemicals from *Solanum xanthocarpum* [18] were tested for their inhibitory action against glucose transporter-4-protein (GLUT4) and regulation of glucose uptake by cancer cells [301]. To this aim, seven retrieved hits from *Solanum xanthocarpum* (stigmasterol glucoside, caffeic acid, apigenin, esculin, scopoletin, lupeol, and solasodine) [302] were downloaded from PubChem database [303]. They belong to different chemical classes of alkaloids, coumarins, flavonoids, steroids, terpenoids, and phenols. These compounds were previously reported to have unique anticancer properties [304–308]. Examinations for their drug likeness and ADME/Tox properties were carried out using Molinspiration [132] and PreADMET v.2 [309], respectively. MD studies were used to test for possible interactions of doxorubicin (a standard anticancer drug) and seven phytochemicals from one end as the ligand and the GLUT4 3D structure from the other end as the receptor, using the PatchDock server [310]. As X-ray structures of this biologically relevant protein are not available, PDB ID: 4PYP [311] was used as a receptor.

Among the seven compounds, only five (solasodine, caffeic acid, esculin, apigenin, and scopoletin) satisfied Lipinski's Rule of Five for oral drug administration. On the other hand, five compounds were predicted to be mutagenic (scopoletin, caffeic acid, esculin, apigenin, and lupeol), while two (stigmasterol glucoside and solasodine) were predicted to be safe.

The highest binding score was produced by PatchDock for stigmasterol glucoside, which was even higher than that for the control ligand (doxorubicin). Stigmasterol glucoside formed hydrogen bond interactions with Gln439, a bump with Asn431, Ile184, and Ala86, and *π*-alkyl bond interactions with Ile184, Ile42, Phe88, Tyr308, Ala86, and Val85. Based on the indicated results, stigmasterol glucoside from *Solanum xanthocarpum* could be a likely therapeutic agent in the treatment of cancer via the inhibition of GLUT4 [301].

As the crystal structure of GLUT4 is not yet resolved, homology modeling studies are conducted to study plausible ligand binding to GLUT4 protein. Thus, a specific focus on selective inhibition of GLUT4 over GLUT1 was conducted [312]. The GLUT4 homology model was built by considering the primary amino acid sequence of GLUT4 as the query. The GLUT4 model was utilized for screening a library of different drug-like compounds. Seventeen molecules were figured out and screened via docking studies for their binding stability, and their cytotoxic effect was studied in four cell lines. Specificity for GLUT4 over GLUT1 was confirmed by analyzing the inhibitor binding with the help of the previously established ATB-BMPA photolabeling assay [313]. The two compounds N-(3-(3-(4-fluorophenyl)propyl)benzyl-3-(2-methoxyphenyl)-N-pyridin-4-ylmethyl)propanamide and (3-((3-((methyl((quinolin-6-ylmethyl)amino)methyl)phenoxy)methyl)piperidin-1-yl) (1,4,5,6-tetrahydrocyclopenta[c]pyrazol-3-yl)methanone were confirmed to bind to GLUT4 in GLUT4-suppressed multiple myeloma cells. This is very similar to the main ritonavir effect on the modulation of peripheral disposal of glucose through GLUT4 inhibition [314].

Table 2 provides the docking studies that were reviewed in this study. It explores the inhibitors and plausible binding sites to the protein targets in the insulin pathway.

## 6. Conclusions

It is now well appreciated that in silico studies present a solid background in the field of chemo/bioinformatics. Dissecting the plausible binding sites of predicted lead compounds to the hub proteins in major metabolic and signaling pathways in the human body makes a hot topic in the field. Herein, coherent pipelines used to predict effector natural ligands and their binding sites to major proteins in the insulin signaling cascade are documented. Priori knowledge included in vitro and in vivo experiments as well as resolved protein structures with/without known inhibitors, which were all uncovered in this review. Altogether, the components of this review round up the picture of what has been conducted so far in the bioinformatics field to enhance the studies of drug discovery in the field of diabetes. We hope that this review will contribute to stimulate further work along these lines.

## Figures and Tables

**Table 1:** Natural molecules that affect insulin signaling pathways.

Natural compounds	Structure	Action	Target
Rutin	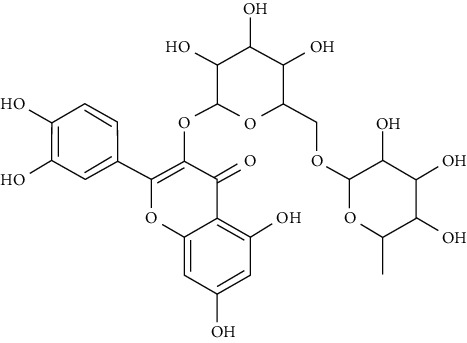	Glycemic control	Improves the insulin receptor activity of kinase (IRK) and the signaling pathway of insulin via increasing the glucose uptake and GLUT4 translocation [69]
Morin	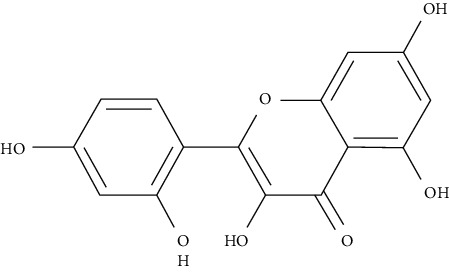	Insulin mimetic	The noncompetitive inhibitor of the bromophenol protein tyrosine phosphatase 1B (PTP1B) escalates phosphorylation of Akt and the insulin receptor (IR). It also controls gluconeogenesis inhibition and glycogen synthesis enhancement [70]
Gallotannins	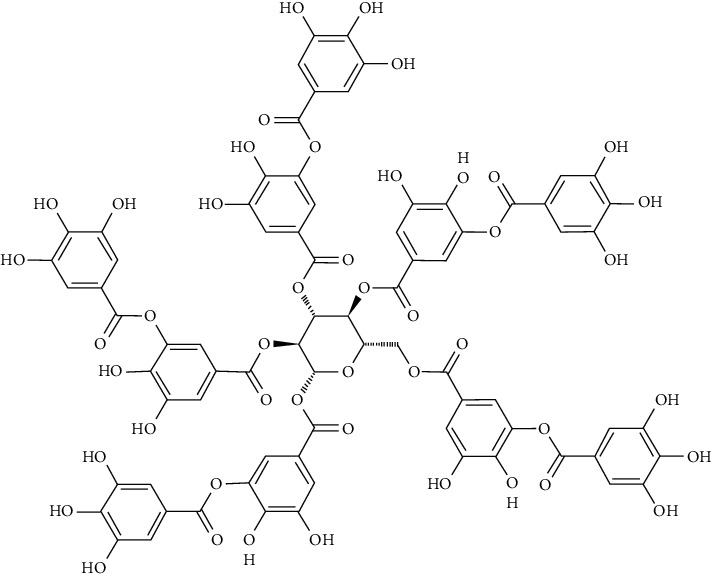	Insulin mimetic	Increases glucose uptake as well as IR and IRS-1 phosphorylation and also mRNA expression of GLUT4 and PI3-kinase in L6 cells [71]
Gallic acid	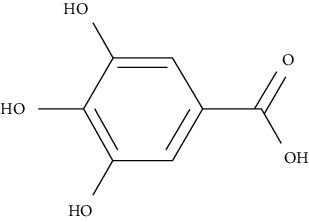	Antidiabetic and antihyperlipidemic	Enhances insulin secretion by conversion of proinsulin to insulin and induces glucose transport through induction of GLUT4 translocation [72]
Oleanolic acid and its derivatives	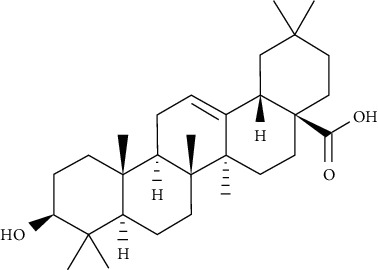	Stimulates insulin sensitivity through the inhibition of PTP1B and other phosphatases activities.	In CHO/hIR cells, it enhances IR and downstream Akt phosphorylation, and in L6 myotubes, it stimulates glucose uptake [73].
Mangiferin	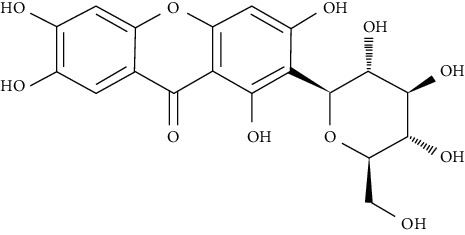	Antidiabetic	Increases the expression of GLUT4 and translocation in muscle L6 myotubes and 3T3-adipocytes cells [74]
Arecoline	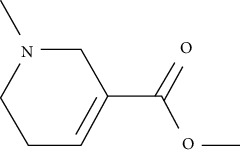	Hypoglycemic	Enhances the translocation of GLUT4 protein via the PPAR*γ* pathway [75]
Berberine	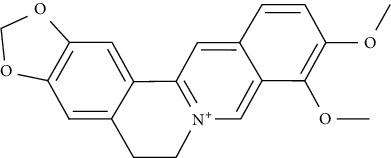		
Vanillic acid	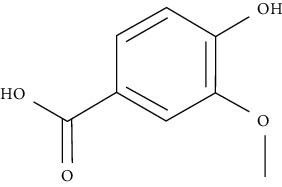		
3*β*-Taraxerol	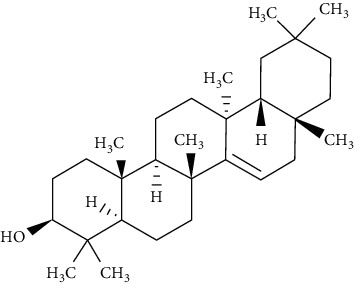	Hypoglycemic	Stimulates glucose transport by facilitating GLUT4 translocation as it activates PI3K and Akt-dependent pathways [76]
Astragalus polysaccharide	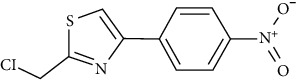	Amelioration of hyperglycemia and insulin resistance	Improves insulin sensitivity by controlling phosphorylation of insulin-induced PKB-Ser473 and translocation of GLUT4 in muscle cells [77]
Cyanidin-3-O-*β*-glucoside	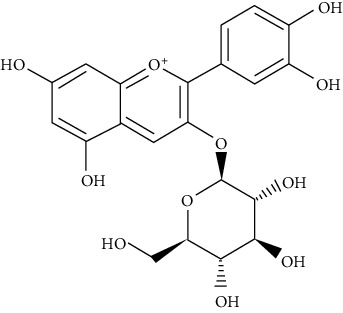	Insulin-like activity	Exerts insulin-like activity by stimulating secretion of adiponectin and translocation of GLUT4, which probably improves the activity of PPAR*γ* [78]
Protocatechuic acid	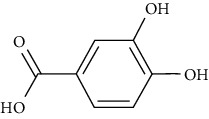		
Daidzein	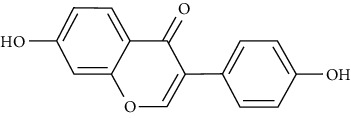	Antidiabetic	In type 2 diabetic mice, it improves glucose homeostasis by enhancing AMPK phosphorylation and GLUT4 protein translocation of muscle cells [79]
Iridoid	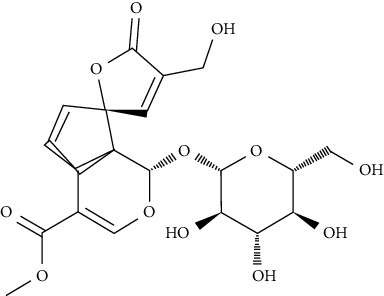	Stimulation in the translocation of GLUT4	In skeletal muscle, it stimulates GLUT4 translocation to cell surface [80]
Catalpol	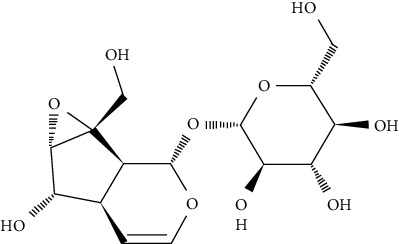		
Specioside	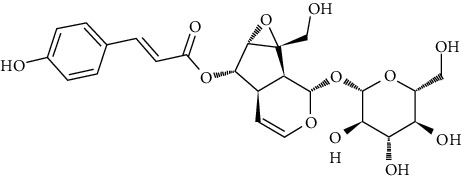		
Verminoside	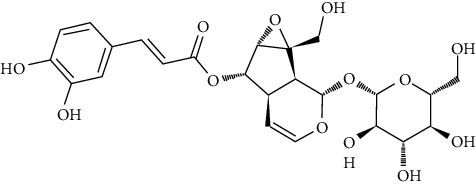		
Lupeol	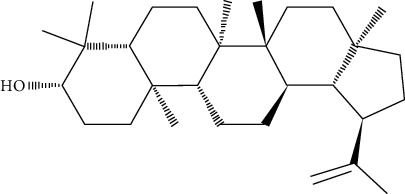	Stimulation of glucose uptake	Stimulates translocation of GLUT4 by activating the RS-1/PI3K/Akt-dependent signaling pathway in L6 cells [81]
Lupeol-trifluoroacetate	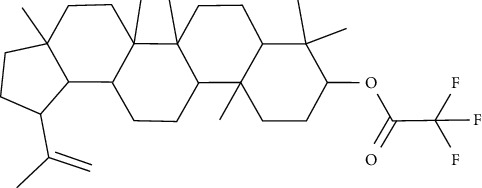		
Palmitic acid	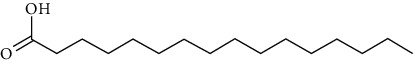	Stimulation of glucose uptake	Enhances glucose uptake in cell line L6 in rat skeletal muscles, through activating the ERK1/2 and Akt pathways [82]
*α*, *β*-Amyrin	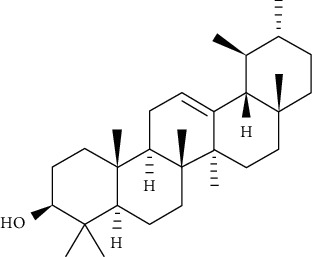 Alpha-amyrin	Antiadipogenic	In 3T3-L1 adipocytes, it increases the expression levels of membrane GLUT4 [83]
	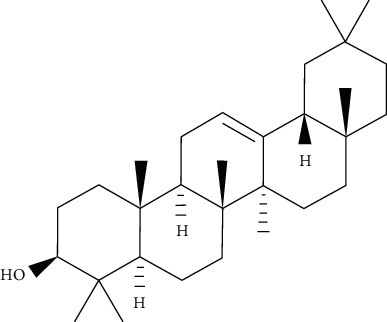 Beta-amyrin		
Ursolic acid	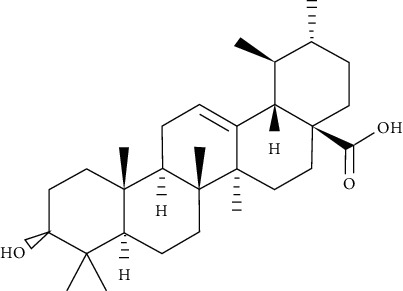	Stimulation of the translocation of GLUT4 and the uptake of glucose	In 3T3-L1 adipocytes, it stimulates the expression of GLUT4 through the PI3K pathway [84]; the combination of ursolic acid along with rosiglitazone enhances the insulin sensitivity by escalating insulin-stimulated IRS-1 tyrosine phosphorylation in the skeletal muscle in diabetic mice [85]
Protocatechuic acid (4-hydroxybenzoic acid)	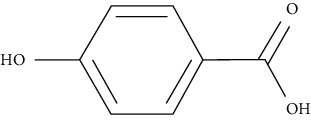	Insulin mimetic	Insulin-like action via activating the AMPK and the INSR/PI3K/Akt pathways. It also stimulates the uptake of glucose through translocation of GLUT4 [86]
Myo-inositol	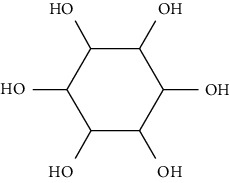	Insulin-sensitizing	In the skeletal muscles of mice, it escalates the translocation of GLUT4 and decreases the levels of postprandial blood glucose [87]. Additionally, it increases GLUT4 levels by activating the AMPK pathway [88]
Naringenin	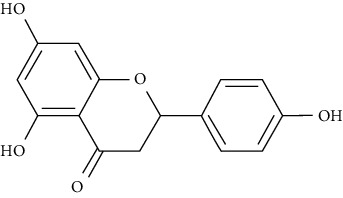	Antihyperglycemic and antihyperlipidemic	Enhances glucose homeostasis in diabetic rats and insulin sensitivity. It also modulated the expressions of GLUT4 protein [89]
Marine collagen peptides	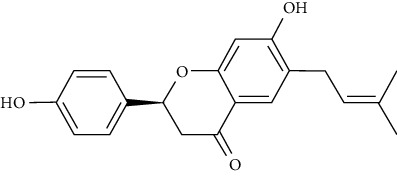	Improvement in the insulin resistance and the glucose metabolism	Enhances the insulin sensitivity via the upregulation of GLUT4 and PPAR*α* expression of diabetic rats [90]
Bavachin		Activating insulin signaling pathway	Improves the uptake of glucose mediated with translocation of GLUT4 by activating the AMPK and Akt pathways in insulin presence or absence [91]
Rosmarinic acid	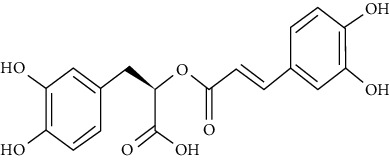	Ameliorates the insulin sensitivity and reduces hyperglycemia	Reduces insulin sensitivity by lowering the expression of PEPCK and elevating the expression of GLUT4 in rats with high-fat diet-induced type 2 diabetes or with streptozotocin (STZ)-induced type 1 diabetes [92]
Dehydroeburicoic acid	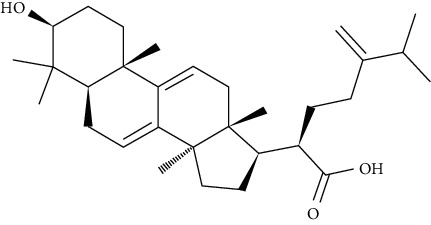	Hypoglycemic	In the skeletal muscle, it increases the membrane levels of GLUT4 and enhances the expressions of skeletal muscle and hepatic AMPK phosphorylation in high-fat diet diabetic mice (HFD) [93]
Baicalin and its metabolites	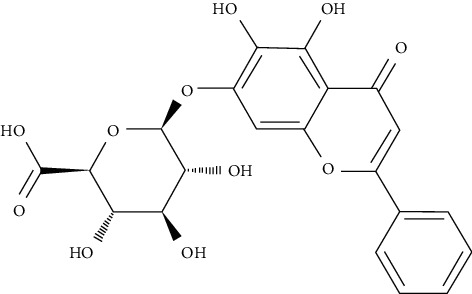	Antihyperglycemic	Suppresses hepatic gluconeogenesis mediated by activating the AMPK and PI3K/Akt signaling pathways [94]
Kazinol B	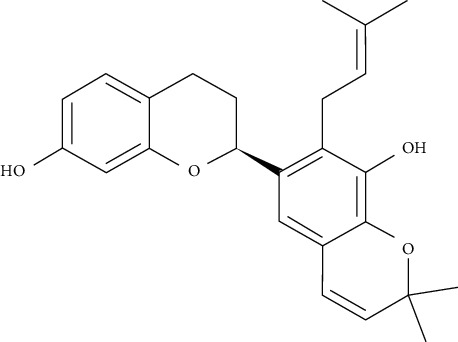	Antidiabetic	Enhances insulin sensitivity by activating AMPK and Akt signaling pathways and by stimulating the adiponectin gene expression and secretion [95]
Octaphlorethol A	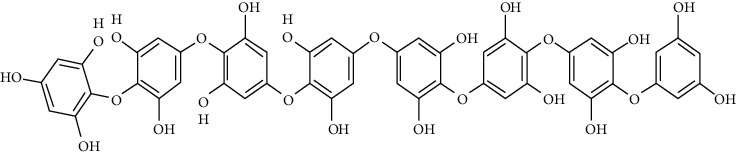	Antidiabetic	In skeletal muscle, it improves the uptake of glucose by elevating the GLUT4 expression by activating the AMPK pathway [96]
Phloridzin	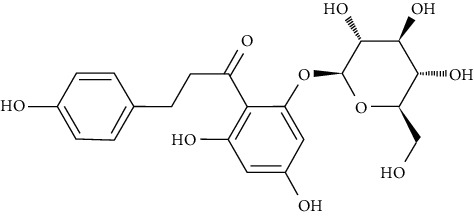	Hypoglycemic	In the liver, it promotes the uptake of glucose by the elevated production of glycogen. Additionally, glucokinase, glucose transporter 2 (GLUT2), IR, and IRS expressions are upregulated [97]
Pterosin A	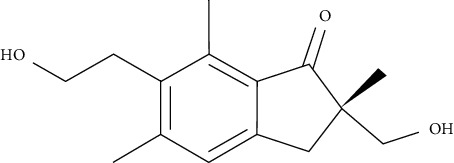	Antidiabetic	Intervenes with GLUT4 translocation, expression of PEPCK, phosphorylation of AMPK, and acetyl-CoA carboxylase, as well as the glycogen synthase kinase-3; decreases glycogen synthase phosphorylation; and increases the intracellular glycogen level [98]
Piceatannol	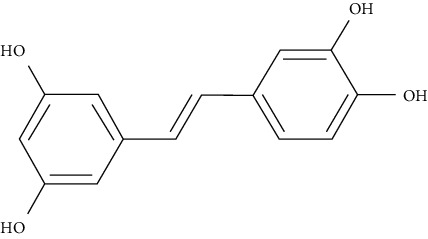	Antidiabetic	In L6 myocytes, it promotes the uptake of glucose, translocation of GLUT4, and phosphorylation of AMPK [99]
Resveratrol	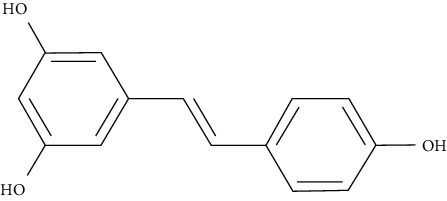	Antidiabetic	Reduces blood insulin levels and adiposity. It also improves translocation of GLUT4 via activation of AMPK as well as the SIRT1 pathway; it also influences the secretion of insulin and concentration of blood insulin by protection of *β*-cells of the pancreatic islets [100–103]
Chlorogenic acid	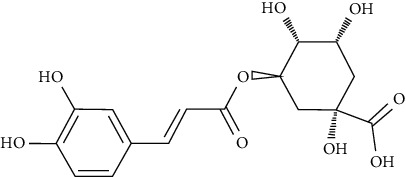	Antidiabetic and antilipidemic	Increases GLUT4 translocation to the PM and triggers AMPK phosphorylation in the skeletal muscle [104–106]
Honokiol	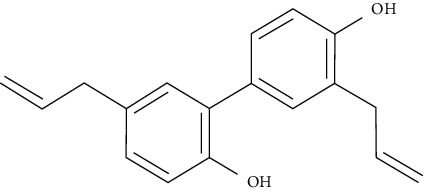	Hypoglycemic	Increases insulin receptor *β*-subunit (IR*β*) phosphorylation as well as the signaling factors of insulin, such as ERK1/2 and Akt. Additionally, it also enhances the translocation of insulin-stimulated GLUT4 and improves the insulin sensitivity by targeting PTP1B [106, 107]
Kaempferol	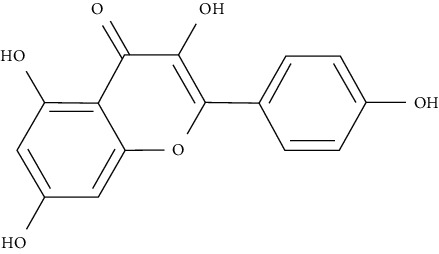	Antidiabetic	Enhances lipolysis and prevents high fatty acid-impaired glucose uptake, AMPK activity, and GLUT4 expression levels in skeletal muscle cells. It also improves peripheral insulin sensitivity and protects against dysfunction of pancreatic *β*-cell [108]
3-Bromo-4,5-bis(2,3-dibromo-4,5-dihydroxybenzyl)-1,2-benzenediol (CYC31)	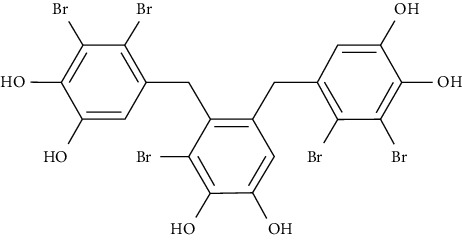	PTP1B inhibitor, activation of insulin signaling	It augments the insulin signaling activity. It also promotes the uptake of 2-NBDG by facilitating translocation of GLUT4 in C2C12 myotubes. Moreover, in C2C12 myotubes, it ameliorates palmitate-induced insulin resistance. Furthermore, it might hinder palmitate-induced insulin resistance and possibly enhance oxidation of fatty acids by inhibiting PTP1B [109]
Carainterol A	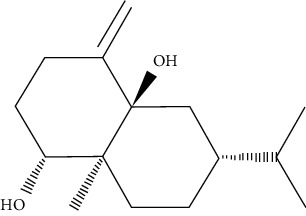	Insulin signaling pathway (regulation of the IRS-1 level)	Increases the pathway sensitivity of insulin based on regulation of the IRS-1 level without influencing translocation of GLUT4 translocation [110]
Bis(2,3-dibromo-4,5dihydroxybenzyl) ether (BDDE)	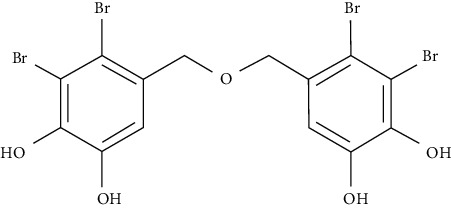	PTP1B inhibitor	In HepG2 cells, it augments the insulin resistance and uptake of glucose. It acts as a PTP1B inhibitor. It also stimulates the signals downstream in insulin signaling pathways such as PI3K, IR*β*, IRS-1/2, and Akt in the db/db mice model [111]
Galangin	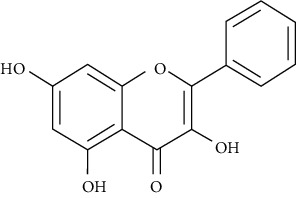	DPP4 inhibitor	Promotes glucose uptake in skeletal muscles [112]
Chrysin	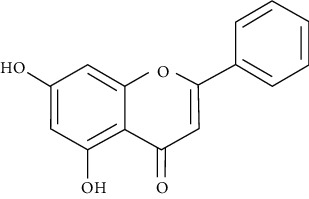	DPP4 inhibitor	Promotes glucose uptake in skeletal muscles [113]

**Table 2:** Inhibitors of protein targets in the insulin signaling pathways, studied via molecular docking, and their plausible binding sites.

Protein	Inhibitors found using docking experiments	Interaction used AA residues
Insulin receptor and PTP-1*β*	IR: herbacetin and sorbifolin [120] IR: ANP [129] PTP1*β*: secoisoresinol, pinoresinol, cedeodarin, and UN608 [129] PTP1B: CYC31 [109] PTP1B: carainterol A [110]	IR-PTP1*β*: Tyr48, and His110 [120] IR: Leu1002, Met1079, and Asp1150 [120] PTP1*β*: Ser1006, Lys1030, Asp1083, Met1079, and Glu1077 [129] PTP1B: Ala217, Arg221, Gly183, and Gln266 [109]

PI3K signaling proteins	PDK1-PI3K: myricetin, quercetin, morin, luteolin, and emodin [138] Akt: sulforaphane and curcumin [138] PIK3-Akt: carainterol A [110] Akt1: 6-0-feruloyl catalpol, 5,7-dihydroxy-8-(3-methyl-2-butenyl) coumarin, isobutrin, MBH-4-flouroracemic, irigenin, junipeginin C, 5,7,4′-trihydroxyflavone-3-O-glucuronide, and ovalitenone [185]. Akt1: 3,5-trisubstituted pyridines, 5-pyrrolopyridinyl-2-thiophenecarboxamides, pyrrolopyrimidine, dihydrothieno and dihydrofuropyrimidines, and isoquinoline–pyridine with oxindole–pyridine derivatives [185]. Akt1: quercetin-7-O-*β*-D-glucopyranoside [200]. Akt-MTOR: 6-methylheptyl sulfate, aegelinol, 2-hydroxynephthenol, iodocionin, (Z)-oct-5-enyl sulfate, osthol [53] Akt: vemurafenib [148]. MTOR: riboflavin [148]. PI3K-Akt-MTOR: wotmannin, MK-2206, LY-294002, mitoxantrone, and rapamycin [148]. Akt1: hesperetin [171]	PDK1: Leu88, Leu159, Ala109, Val143, Tyr161, Ala162, Ala162, Leu212, Ser160, Glu209, and Thr222 [138] PIK3: Pro810, Trp812, Tyr867, Ile 881, Val882, Ala885, Ile879, met 953, and Phe961 [138] Akt1: Leu156, Val164, Ala177, Met227, Tyr229, Ala230, Phe236, Phe237, Met281, Tyr437, Phe438, and Phe442; polar Thr211, Asp279, Thr291, Thr435, and Thr443 residues; negatively charged Glu228, Glu234, Glu278, Asp292, and Asp439 residues; positively charged Lys158, Lys179, and Arg241 [185]. Akt: Ala230, Glu226, Glu234, Tyr272, Val164, Ala177, Ilu800, Thr291, and Met28 [148] MTOR: Val882 and Lys890 [148]. Akt1: Gln79, Thr211, and Leu210 [171]. PI3K*α* site A: Lys802, Asp810, Asp933, and Val851 [152]. PI3K*α* site B: Val851 and Gln859 [152]. PI3K*γ* site A: Lys833 [152]. PI3K*γ* site B: Lys833 and ASP844 [152]. PI3K–C2*β*: Val1115 [152]. P70S6K1: Glu143 and Lys123 [152]. mTOR site A: Lys-asp pair [152] mTOR site B, backbone nucleotide loop [152]. PI3K*γ*: Met804, Ser806, Trp812, Ile831, Lys833, Tyr867, Ile879, Asp950, Asn951, Met953, Ile963, and Asp964 [159]. Akt: Residues Leu156, Gly157, Val164, Ala177, Thr211, Met227, Glu228, Ala230, Glu234, Met281, Thr291, Asp292, Phe438, and Phe442 [159]. mTOR:: Leu2185, Glu2190, Asp2195, Tyr2225, Ile2237, Gly2238, Trp2239, Val2240, Met2345, Ile2356, and Asp2357 [159]. PI3K: His39, Phe87, Phe88, Phe111, Phe112, Asn114, Met115, Phe116, Tyr166, Arg170, Tyr184, Asp185, Val186, Leu187, Gln203, Leu206, Lys210, Phe211, Trp259, Gly260, Tyr262, His263, Phe265, Ser266, Leu267, Glu269, Ala270, Trp283, Leu286, Pro287, Phe288, Val289, Thr290, Ser291, Leu292, and Ala293 Phe294 [219]. Akt: Ser205 [223]

Further proteins effecting insulin signaling	RAC1: subsets of flavonoids, morpholines, and imidazoles [251] RAC1-HSP90-EGFR-CDH1-COPS2: rhein [259] SRAC1: 4F rhein derivative, NSC23766 [274]	RAC1: Asp65-arg-66-Arg68-Pro69 and Ala95-lys96-pro99-glu100-his103-his104 [251].

PTEN	Naringin [279]	Cys124, Arg130, His93, Gly127, Asp92, Gln171, Ala126, Lys125, and Lys 128 [287]. Tyr16, Asp24, Arg47, Gly44, Val45, Asp92, His93, Ala126, Lys128, Asp162, Lys164, Asp326, Lys330.Tyr16, Val45, Asp92, and Ala126 [299]. Tyr16, Val45, Ala126, and Asp92 [300].

GLUT4	Stigmasterol glucoside [301]. N-(3-(3-(4-Fluorophenyl)propyl)benzyl-3-(2-methoxyphenyl)-N-pyridin-4-ylmethyl)propanamide and (3-((3-((methyl((quinolin-6-ylmethyl)amino)methyl)phenoxy)methyl)piperidin-1-yl) (1,4,5,6-tetrahydrocyclopenta[c]pyrazol-3-yl)methanone [312]	Gln439, Ala86, Ile184, Asn431, Phe88, Ile42, Tyr308, Val85, Ala86, and Ile184 [301]. Leu185, Ile42, Phe307, Asn427, Asn176, and Trp404 [312]. Ile42, Leu185, Phe38, Phe307, Asn427, and Trp404 [312].

## Data Availability

The data used to support the findings of this study are available from the corresponding author upon request.

## Supplementary Materials

Supplementary Materials Structural evidences for the proteins involved in the insulin signaling cascade.
