# Supplementary material for: Drug Discovery of Plausible Lead Natural Compounds That Target the Insulin Signaling Pathway: Bioinformatics Approaches
Source: Evid Based Complement Alternat Med. 2022 Mar 20;2022:2832889. doi: 10.1155/2022/2832889 (PMC8958086; doi:10.1155/2022/2832889)
Supplement: Supplementary Materials — Structural evidences for the proteins involved in the insulin signaling cascade. [file 2832889.f1.docx]

**Supplementary Material**

**Structural Evidences for the Proteins involved in the Insulin Signaling Cascade**

We used UniProt database to uncover structural evidences for proteins involved in insulin signaling cascade in human [320]. In this respect, we also shed light on the structures with inhibitors involved. Figure S1 summarizes selected resolved protein structures in the insulin signaling pathway.


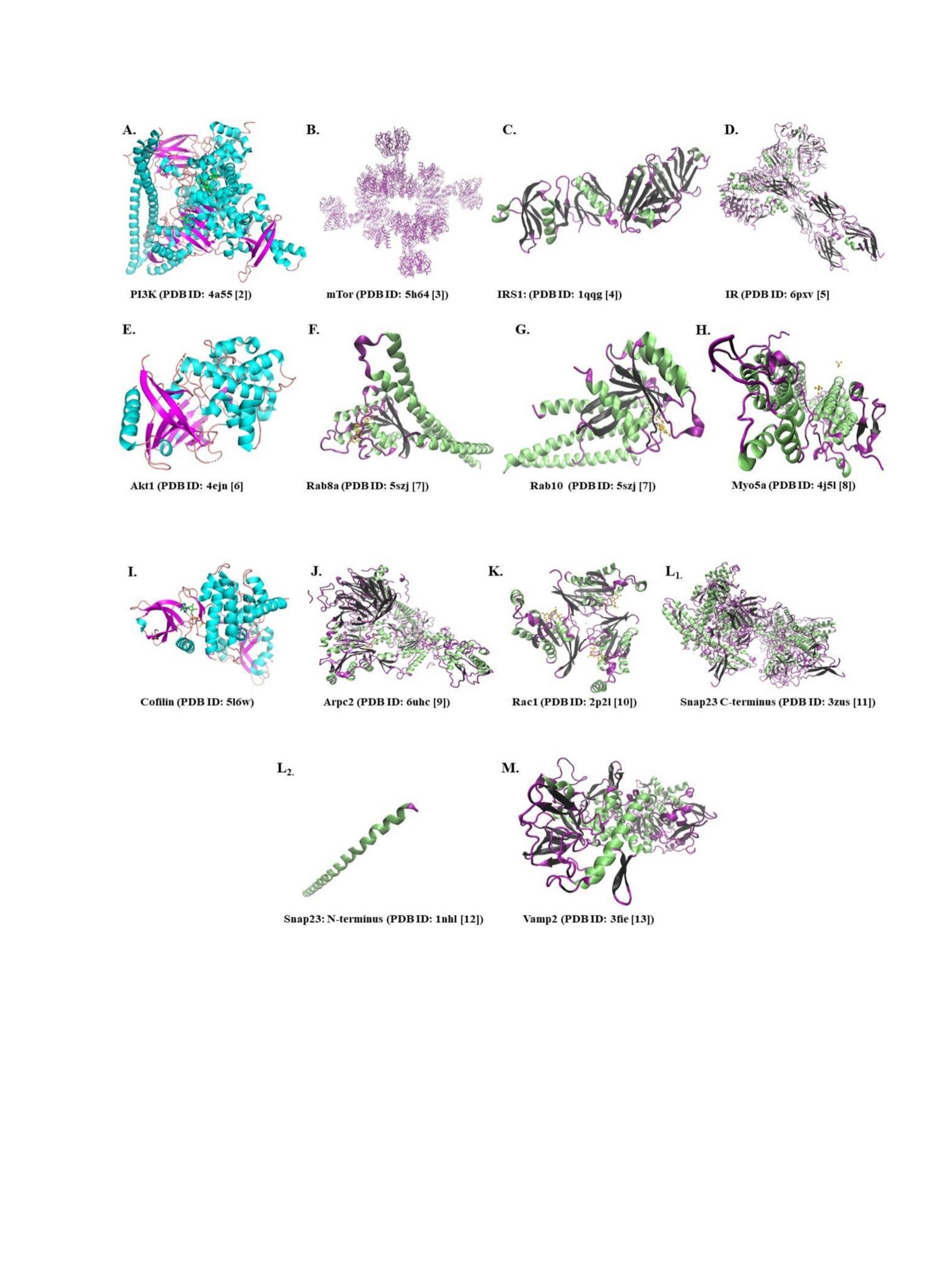


**Figure S1.** Exemplary resolved protein structures in the insulin signaling pathway, retrieved from RCSB Protein Data Bank [206]

1. **Phosphoinositide 3-kinase, PI3K** (<https://www.uniprot.org/uniprot/P27986>)

PI3K is a 724 amino acid sequence. It is composed of alternating alpha-helices and beta-sheets, where the alpha-helix secondary structure dominates. The following domains exist from the N-terminus to the C-terminus: and SH3 domain (3-79 aa), a Rho-GAP domain (113-301 aa), and two SH2 domains (333-428, 624-718 aa, respectively). Several crystallographic evidences have been resolved for the PI3K protein that cover several patches in the protein structure. Of these structures, 5M6U is the structure that resolves the full sequence at a 2.85 A resolution [321].

The 4A55 structure resolves the target protein complexed to the inhibitor PIK-108 [242]. Additional to the ATP-binding pocket, a second PIK-108 binding site was observed in the kinase C-lobe. The allosteric regulation of the kinase domain by induces a global conformational change in p110α.

The PDB structure 4JPS unravels the NVP-BYL719 as a potent and selective inhibitor for PI3K. Indeed, many crystal structures were resolved that target the PI3K for inhibition (PDB IDs: 4waf, 4i2y, 4i23, 4i1b, 4zop, 6pyu, 6pyr, 5xgi, 5vlr, 5ukj, 5ul1, 5ubt, 5m6u, 5itd) [154, 321-327].

Crystal structures of PI3K bound to inhibitors show that ligands bind in the ATP-binding site, located in the kinase domain between the N-terminal and C-terminal lobes [328]. A hinge region that provides hydrogen bond donor and acceptor sites is found at the base of the ATP-binding cavity. A hydrophobic pocket is also located in this domain [167]. PI3Kα-selective inhibition can be achieved by interacting with the p110α-specific amino acid Gln859 in a non-conserved area called region I [154, 238-240].

1. **Mammalian target of rapamycin, mTOR** (<https://www.uniprot.org/uniprot/P42345>)

mTOR is a 2549 amino acid sequence. Several crystal structures were resolved that cover patches from the whole sequence of this protein. The protein is composed of, from the N-terminus to the C-terminus, 32 HEAT repeats that range from amino acids 16-1345. In the 1-651 amino acid range, an interaction with the NBN domain was experimentally determined [329]. Sixteen TRP repeats range from amino acids 1346-2005. A FAT domain is located within three discontinuous subdomains of alpha-helical TPR repeats and a single subdomain of HEAT repeats. The four domains fold serially and form a C-shaped tight coil that holds onto the kinase domain [168]**.**  The PI3K/PI4K domain is located in the 2182-2516 amino acid sequence. The PI3K/PI4K domain was found to be active with a restricted catalytic center [168]. Lastly, a FATC domain is located in the 2517-2549 amino acid range.

Many structures were resolved that target the inhibition of mTOR (PDB IDs: 5wbu, 5wbh, 6bcx, 5wby, 6m4u, 6m4w, 4jsp, 4jt6, 5gpg, 2gaq, 2npu, 3fap, 4drh, 4dri, 4drj, 4fap) [147, 330-335].

1. **Insulin receptor substrate 1, IRS1** (<https://www.uniprot.org/uniprot/P35568>)

IRS1 is composed of 1242 amino acids. Alternating beta sheets and alpha helices exist at the N-terminal region. Two domains exist at the N-terminus, the PH domain (12-115 amino acids) and the IRS-type PTB domain (160-264 amino acids) “PROSITE-ProRule Annotation” [336]. Nine interspersed YXXM motifs span the length of the protein, from amino acids 465 to 1015. Several structural evidences were resolved that uncover crucial functioning of the IRS-1 (1IRS, 1K3A, 1QQG, 2Z8C, 5U1M, 6BNT) [337-341] One of the crystal structures resolved the binding of IRS-1 to inhibitors [342].

1. **Insulin receptor, IR** (<https://www.uniprot.org/uniprot/P06213>)

IR is 1382 amino acid in length. Several structural evidences were resolved for the protein. Most targeted the 1005-1310 region (e.g., 1GAG, 1I44, 1IR3, 1IRK, 1P14, 1RQQ, 2AUH, 2B4S), where the protein kinase domain is located [336]. The 1GAG structural evidence, for example, covered the binding of insulin receptor to inhibitors [343]. A synthetic inhibitor was found to be a high affinity competitive inhibitor against both nucleotide and peptide substrates. A crystal structure of this inhibitor bound to the tyrosine kinase domain of the IR revealed that the inhibitor participates in the octahedral coordination of an Mg2+ in the active site [343]. Other domains in the insulin receptor from the N-terminus include three Fibronectin type-III domains (1, 2, and 3, 624-947 aa) [336].

Several structures were additionally resolved to study the binding of inhibitors. These include the following PDB IDs (2AUH, 2Z8C, 3EKK, 3EKN, 3ETA, 4IBM, 5E1S, 5HHW) [342, 344-350].

1. **Protein kinase B, PKB/Akt1** (<https://www.uniprot.org/uniprot/P31749>)

Akt1 is 480 amino acid length. From the N-terminus to C-terminus, it is composed of the PH domain (5-108 amino acids), the protein kinase domain (150-408 amino acids), and the AGC-kinase C-terminal domain (409-480 amino acids) “PROSITE-PRoRule annotation” [336]. Regions positioned at 14-19 amino acids, and 23-25 amino acids confer the Inositol-(1,3,4,5)-tetrakisphosphate binding. Inhibitor binding resolved to occupy the 228-230 region. Several structural evidences resolved the nearly full protein structure (spanning the 2-446 amino acid sequence, e.g., PDB ID: 4EJN, 5KCV, 6HHF, 6HHG, 6HHH, 6HHI, 6HHJ, 6S9W, 6S9X) [351-354]. All of the aforementioned structures study the binding of inhibitors to Akt1 protein.

1. **Ras-related protein 8a, Rab8a** (<https://www.uniprot.org/uniprot/P61006>)

Rab8a is 207 amino acids in length, with alternating alpha-helices an beta-sheets. Most structural evidences cover most of the structure. One structure covers the full length (PDB ID: 5SZI [355]).

1. **Ras-related protein-10, Rab10** (<https://www.uniprot.org/uniprot/P61026>)

The Rab10 protein is 200 amino acid length, with alternating alpha helices and beta sheets. Only two structural evidences were resolved for this protein (PDB ID: 5LPN, 5SZJ [355]). 5SZJ resolves the full protein structure.

1. **Myosin VA, Myo5a** (<https://www.uniprot.org/uniprot/Q9Y4I1>)

Myo5a is 1855 amino acids in length. From the N-terminus to the C-terminus, it comprises several domains, the myosin N-terminal SH3-like domain (8-60 aa), the myosin motor domain (69-763 aa), six IQ subdomains (1-6: 766-914 aa), and a dilute domain (1534-1810 aa) “PROSITE” [336]. The resolved crystal structures cover the C-terminal region of the protein, some spanning the 1275-1297 aa region (PDB ID: 4D07) [356]. Other structures cover the 1448-1855 region (PDB ID: 4J5L, 4LLI, 4LX1, 4LX2, 5JCY, 5JCZ) [357-359].

1. **Cofilin** ( <https://www.uniprot.org/uniprot/P23528>)

Cofilin is 166 amino acids in length, with an ADF-H domain (4-153 aa) “ProSite ProRule annotation” [336]. All resolved structures covered the 1-166 amino acid span. Whereas most structural evidences were based on electron microscopy, NMR and X-ray structures were also resolved. T with the most reasonable resolutions belonged to the X-ray structure with the PDB ID: 5L6W (structure not yet published). Other crystallographic evidences are the PDB IDs: 4BEX and 5HVK [360, 361]

1. **Arp2/3 (Arpc2)** (<https://www.uniprot.org/uniprot/O15144>)

This protein is 300 aa in length. Three structures were resolved via CryoEM (PDB ID: 6UHC, 6YW6, 6YW7) [362, 363]

1. **Ras-related C3 botulinum toxin substrate 1, Rac1**

(<https://www.uniprot.org/uniprot/P63000>)

Rac1 is 192 amino acids in length, with alternating alpha-helices and beta-sheets. Many crystallographic and NMR evidences were resolved for the protein. The one of choice here is PDB ID: 2p2l. Herein, a Rac1-GDP-Zn complex has been crystallized. Rac1 was shown to coordinate Zn atoms in a tetrahedral fashion, using the biologically relevant switch I and switch II regions [364]

The PDB ID: 1G4U represents the binding of an effector to Rac1. This binding stabilizes a four-helix bundle that makes extensive contacts with the Switch I and Switch II regions of the GTPase [272].

The PDB ID: 1HE1 represents the crystal structure of the complex between the GAP domain of the Pseudomonas aeruginosa ExoS toxin and human Rac.

The ExoS downregulates Rac using an arginine finger to stabilize the transition state of the GTPase reaction *[365]*.

The PDB ID 2H7V represents the co-crystal structure of YpkA-Rac1. YpkA inhibits nucleotide exchange in Rac1 and RhoA. In the crystal structure of YpkA-Rac1 complex, YpkA was found to have a Rac1 binding domain that mimics guanidine nucleotide dissociation inhibitors (GDIs) of the Rho GTPases [366].

In the crystal structure with PDB ID: 56NO, the guanine nucleotide exchange factor DOCK5 is allosterically inhibited of by C21, a small molecule. This molecule blocks the catalytic activity of the DHR2 domain of DOCK5 in a non-competitive fashion [367].

1. **Synaptosomal-associated protein 23, Snap23**

(<https://www.uniprot.org/uniprot/O00161>)

SNAP23 is 211 amino acids long. Two t-SNARE coied coil homology domains exist in this protein, 1 and 2 “PROSITE-ProRule” [336]. Two crystal structures were resolved for this protein, each one targeting one of the two domains. The PDB ID: 1NHL targets the N-terminal coiled coil (23-76 aa) [368]

The PDB ID: 3ZUS targets the C-terminal sequence (150-211 aa) [369]

1. **Vesicle-associated membrane protein 2, Vamp2**

(<https://www.uniprot.org/uniprot/P63027>)

The Vamp2 protein is 116 aa n length. However, the resolved crystal structures only concentrated on the 28-60 central amino acids (PDB IDs: 3FIE, 3FII, 3RK2, 3RK3, 3RL0) [370, 371] with the emphasis of *Clostredium botulinum* toxins targeting the structures [370] and inducing the cleavage of neuronal proteins responsible for neurotransmitter release.

Two substrate-based inhibitors bind to the protein, VAMP 22-58/Gln58D-cysteine and 27-58/Gln58D-cysteine. The cysteine sulfur of the inhibitors interacts with the zinc and exists as sulfinic acid in the inhibitor VAMP 27-58/Gln58D-cysteine. Arg133 and Arg171 form parts of two secondary binding sites and are crucial for substrate catalysis [370].

For other proteins in the insulin signaling cascade (Munc18c, or stxbp3; Syntaxin; Glut4; Rab13; Sec16A), after a careful searching of the UniProt database, and to the best of our knowledge, no structural evidences were solved for humans so far. Thus, the need for homology modeling, using the present crystal structures of close species, is needed to investigate plausible effector-receptor interactions in our studied proteins.

[320] Bateman A, Martin MJ, Orchard S, Magrane M, Agivetova R, Ahmad S, Alpi E, Bowler-Barnett EH, Britto R, Bursteinas B, Bye-A-Jee H, Coetzee R, Cukura A, Da Silva A, Denny P, Dogan T, Ebenezer T, Fan J, Castro LG, Garmiri P, Georghiou G, Gonzales L, Hatton-Ellis E, Hussein A, Ignatchenko A, Insana G, Ishtiaq R, Jokinen P, Joshi V, Jyothi D, Lock A, Lopez R, Luciani A, Luo J, Lussi Y, Mac-Dougall A, Madeira F, Mahmoudy M, Menchi M, Mishra A, Moulang K, Nightingale A, Oliveira CS, Pundir S, Qi GY, Raj S, Rice D, Lopez MR, Saidi R, Sampson J, Sawford T, Speretta E, Turner E, Tyagi N, Vasudev P, Volynkin V, Warner K, Watkins X, Zaru R, Zellner H, Bridge A, Poux S, Redaschi N, Aimo L, Argoud-Puy G, Auchincloss A, Axelsen K, Bansal P, Baratin D, Blatter MC, Bolleman J, Boutet E, Breuza L, Casals-Casas C, de Castro E, Echioukh KC, Coudert E, Cuche B, Doche M, Dornevil D, Estreicher A, Famiglietti ML, Feuermann M, Gasteiger E, Gehant S, Gerritsen V, Gos A, Gruaz-Gumowski N, Hinz U, Hulo C, Hyka-Nouspikel N, Jungo F, Keller G, Kerhornou A, Lara V, Le Mercier P, Lieberherr D, Lombardot T, Martin X, Masson P, Morgat A, Neto TB, Paesano S, Pedruzzi I, Pilbout S, Pourcel L, Pozzato M, Pruess M, Rivoire C, Sigrist C, Sonesson K, Stutz A, Sundaram S, Tognolli M, Verbregue L, Wu CH, Arighi CN, Arminski L, Chen CM, Chen YX, Garavelli JS, Huang HZ, Laiho K, McGarvey P, Natale DA, Ross K, Vinayaka CR, Wang QH, Wang YQ, Yeh LS, Zhang J, UniProt C. UniProt: the universal protein knowledgebase in 2021. Nucleic Acids Research, 2021; 49: D480-D489.

[321] Erra M, Taltavull J, Greco A, Bernal FJ, Caturla JF, Gracia J, Dominguez M, Sabate M, Paris S, Soria S, Hernandez B, Armengol C, Cabedo J, Bravo M, Calama E, Miralpeix M, Lehner MD. Discovery of a Potent, Selective, and Orally Available PI3K delta Inhibitor for the Treatment of Inflammatory Diseases. Acs Medicinal Chemistry Letters, 2017; 8: 118-123.

[322] Hoegenauer K, Soldermann N, Stauffer F, Furet P, Graveleau N, Smith AB, Hebach C, Hollingworth GJ, Lewis I, Gutmann S, Rummel G, Knapp M, Wolf RM, Blanz J, Feifel R, Burkhart C, Zecri F. Discovery and Pharmacological Characterization of Novel Quinazoline-Based PI3K Delta-Selective Inhibitors. Acs Medicinal Chemistry Letters, 2016; 7: 762-767.

[323] Qin LY, Ruan ZM, Cherney RJ, Dhar TGM, Neels J, Weigelt CA, Sack JS, Srivastava AS, Cornelius LAM, Tino JA, Stefanski K, Gu XM, Xie J, Susulic V, Yang XX, Yarde-Chinn M, Skala S, Bosnius R, Goldstein C, Davies P, Ruepp S, Salter-Cid L, Bhide RS, Poss MA. Discovery of 7-(3-(piperazin-1-yl)phenyl)pyrrolo 2,1-f 1,2,4 triazin-4-amine derivatives as highly potent and selective PI3K delta inhibitors. Bioorganic & Medicinal Chemistry Letters, 2017; 27: 855-861.

[324] Lu YP, Knapp M, Crawford K, Warne R, Elling R, Yan K, Doyle M, Pardee G, Zhang L, Ma S, Mamo M, Ornelas E, Pan Y, Bussiere D, Jansen J, Zaror I, Lai A, Barsanti P, Sim J. Rationally Designed PI3K alpha Mutants to Mimic ATR and Their Use to Understand Binding Specificity of ATR Inhibitors. Journal of Molecular Biology, 2017; 429: 1684-1704.

[325] Liu QJ, Shi Q, Marcoux D, Batt DG, Cornelius L, Qin LY, Ruan ZM, Neels J, Beaudoin-Bertrand M, Srivastava AS, Li L, Cherney RJ, Gong H, Watterson SH, Weigelt C, Gillooly KM, McIntyre KW, Xie JH, Obermeier MT, Fura A, Sleczka B, Stefanski K, Fancher RM, Padmanabhan S, Thatipamula RP, Kundu I, Rajareddy K, Smith R, Herman JK, Xing DZ, Fan JS, Levesque PC, Ruan Q, Pitt S, Zhang R, Pedicord D, Pan J, Yarde M, Lu H, Lippy J, Goldstine C, Skala S, Rampulla RA, Mathur A, Gupta A, Arunachalam PN, Sack JS, Muckelbauer JK, Cvijic ME, Salter-Cid LM, Bhide RS, Poss MA, Hynes J, Carter PH, Macor JE, Ruepp S, Schieven GL, Tino JA. Identification of a Potent, Selective, and Efficacious Phosphatidylinositol 3-Kinase delta (P13K delta) Inhibitor for the Treatment of Immunological Disorders. Journal of Medicinal Chemistry, 2017; 60: 5193-5208.

[326] Fradera X, Methot JL, Achab A, Christopher M, Altman MD, Zhou H, McGowan MA, Kattar SD, Wilson K, Garcia Y, Augustin MA, Lesburg CA, Shah S, Goldenblatt P, Katz JD. Design of selective PI3K delta inhibitors using an iterative scaffold-hopping workflow. Bioorganic & Medicinal Chemistry Letters, 2019; 29: 2575-2580.

[327] Zhao YL, Zhang X, Chen YY, Lu SY, Peng YF, Wang X, Guo CL, Zhou AW, Zhang JM, Luo Y, Shen QC, Ding J, Meng LH, Zhang J. Crystal Structures of PI3K alpha Complexed with PI103 and Its Derivatives: New Directions for Inhibitors Design. Acs Medicinal Chemistry Letters, 2014; 5: 138-142.

[328] Yuan TL, Cantley LC. PI3K pathway alterations in cancer: variations on a theme. Oncogene, 2008; 27: 5497-5510.

[329] Wang JQ, Chen JH, Chen YC, Chen MY, Hsieh CY, Teng SC, Wu KJ. Interaction between NBS1 and the mTOR/Rictor/SIN1 Complex through Specific Domains. Plos One, 2013; 8.

[330] Yang HJ, Jiang XL, Li BR, Miller M, Yang A, Dhar A, Pavletich NP. Mechanisms of mTORC1 activation by RHEB and inhibition by PRAS40. Nature, 2017; 552: 368-+.

[331] Wu HD, Kikuchi M, Dagliyan O, Aragaki AK, Nakamura H, Dokholyan NV, Umehara T, Inoue T. Rational design and implementation of a chemically inducible heterotrimerization system. Nature Methods, 2020; 17: 928-+.

[332] Lee SY, Lee H, Lee HK, Lee SW, Ha SC, Kwon T, Seo JK, Lee C, Rhee HW. Proximity-Directed Labeling Reveals a New Rapamycin-Induced Heterodimer of FKBP25 and FRB in Live Cells. Acs Central Science, 2016; 2: 506-516.

[333] Leone M, Crowell KJ, Chen JH, Jung DW, Chiang GG, Sareth S, Abraham RT, Pellecchia M. The FRB domain of mTOR: NMR solution structure and inhibitor design. Biochemistry, 2006; 45: 10294-10302.

[334] Veverka V, Crabbe T, Bird I, Lennie G, Muskett FW, Taylor RJ, Carr MD. Structural characterization of the interaction of mTOR with phosphatidic acid and a novel class of inhibitor: compelling evidence for a central role of the FRB domain in small molecule-mediated regulation of mTOR. Oncogene, 2008; 27: 585-595.

[335] Liang J, Choi J, Clardy J. Refined structure of the FKBP12-rapamycin-FRB ternary complex at 2.2 angstrom resolution. Acta Crystallographica Section D-Structural Biology, 1999; 55: 736-744.

[336] Sigrist CJA, De Castro E, Langendijk-Genevaux PS, Le Saux V, Bairoch A, Hulo N. ProRule: a new database containing functional and structural information on PROSITE profiles. Bioinformatics, 2005; 21: 4060-4066.

[337] Zhou MM, Huang BH, Olejniczak ET, Meadows RP, Shuker SB, Miyazaki M, Trub T, Shoelson SE, Fesik SW. Structural basis for IL-4 receptor phosphopeptide recognition by the IRS-1 PTB domain. Nature Structural Biology, 1996; 3: 388-393.

[338] Favelyukis S, Till JH, Hubbard SR, Miller WT. Structure and autoregulation of the insulin-like growth factor 1 receptor kinase. Nature Structural Biology, 2001; 8: 1058-1063.

[339] Dhe-Paganon S, Ottinger EA, Nolte RT, Eck MJ, Shoelson SE. Crystal structure of the pleckstrin homology-phosphotyrosine binding (PH-PTB) targeting region of insulin receptor substrate 1. Proceedings of the National Academy of Sciences of the United States of America, 1999; 96: 8378-8383.

[340] Cai WK, Sakaguchi M, Kleinridders A, Gonzalez-Del Pino G, Dreyfuss JM, O'Neill BT, Ramirez AK, Pan H, Winnay JN, Boucher J, Eck MJ, Kahn CR. Domain-dependent effects of insulin and IGF-1 receptors on signalling and gene expression. Nature Communications, 2017; 8.

[341] Choi E, Kikuchi S, Gao HS, Brodzik K, Nassour I, Yopp A, Singal AG, Zhu H, Yu HT. Mitotic regulators and the SHP2-MAPK pathway promote IR endocytosis and feedback regulation of insulin signaling. Nature Communications, 2019; 10.

[342] Katayama N, Orita M, Yamaguchi T, Hisamichi H, Kuromitsu S, Kurihara H, Sakashita H, Matsumoto Y, Fujita S, Niimi T. Identification of a key element for hydrogen-bonding patterns between protein kinases and their inhibitors. Proteins-Structure Function and Bioinformatics, 2008; 73: 795-801.

[343] Parang K, Till JH, Ablooglu AJ, Kohanski RA, Hubbard SR, Cole PA. Mechanism-based design of a protein kinase inhibitor. Nature Structural Biology, 2001; 8: 37-41.

[344] Depetris RS, Hu JJ, Gimpelevich I, Holt LJ, Daly RJ, Hubbard SR. Structural basis for inhibition of the insulin receptor by the adaptor protein Grb14. Molecular Cell, 2005; 20: 325-333.

[345] Chamberlain SD, Wilson JW, Deanda F, Patnaik S, Redman AM, Yang B, Shewchuk L, Sabbatini P, Leesnitzer MA, Groy A, Atkins C, Gerding R, Hassell AM, Lei HS, Mook RA, Moorthy G, Rowand JL, Stevens KL, Kumar R, Shotwell JB. Discovery of 4,6-bis-anilino-1H-pyrrolo 2,3-d pyrimidines: Potent inhibitors of the IGF-1R receptor tyrosine kinase. Bioorganic & Medicinal Chemistry Letters, 2009; 19: 469-473.

[346] Chamberlain SD, Redman AM, Wilson JW, Deanda F, Shotwell JB, Gerding R, Lei HS, Yang B, Stevens KL, Hassell AM, Shewchuk LM, Leesnitzer MA, Smith JL, Sabbatini P, Atkins C, Groy A, Rowand JL, Kumar R, Mook RA, Moorthy G, Patnaik S. Optimization of 4,6-bis-anilino-1H-pyrrolo 2,3-d pyrimidine IGF-1R tyrosine kinase inhibitors towards JNK selectivity. Bioorganic & Medicinal Chemistry Letters, 2009; 19: 360-364.

[347] Patnaik S, Stevens KL, Gerding R, Deanda F, Shotwell JB, Tang J, Hamajima T, Nakamura H, Leesnitzer MA, Hassell AM, Shewchuck LM, Kumar R, Lei HS, Chamberlain SD. Discovery of 3,5-disubstituted-1H-pyrrolo 2,3-b pyridines as potent inhibitors of the insulin-like growth factor-1 receptor (IGF-1R) tyrosine kinase. Bioorganic & Medicinal Chemistry Letters, 2009; 19: 3136-3140.

[348] Anastassiadis T, Duong-Ly KC, Deacon SW, Lafontant A, Ma HC, Devarajan K, Dunbrack RL, Wu JH, Peterson JR. Highly Selective Dual Insulin Receptor (IR)/Insulin-like Growth Factor 1 Receptor (IGF-1R) Inhibitor Derived from an Extracellular Signal-regulated Kinase (ERK) Inhibitor. Journal of Biological Chemistry, 2013; 288: 28068-28077.

[349] Sanderson MP, Apgar J, Garin-Chesa P, Hofmann MH, Kessler D, Quant J, Savchenko A, Schaaf O, Treu M, Tye H, Zahn SK, Zoephel A, Haaksma E, Adolf GR, Kraut N. BI 885578, a Novel IGF1R/INSR Tyrosine Kinase Inhibitor with Pharmacokinetic Properties That Dissociate Antitumor Efficacy and Perturbation of Glucose Homeostasis. Molecular Cancer Therapeutics, 2015; 14: 2762-2772.

[350] Stauffer F, Cowan-Jacob SW, Scheufler C, Furet P. Identification of a 5- 3-phenyl-(2-cyclic-ether)-methylether -4-aminopyrrolo 2,3-d pyrimidin e series of IGF-1R inhibitors. Bioorganic & Medicinal Chemistry Letters, 2016; 26: 2065-2067.

[351] Lapierre JM, Eathiraj S, Vensel D, Liu YB, Bull CO, Cornell-Kennon S, Iimura S, Kelleher EW, Kizer DE, Koerner S, Makhija S, Matsuda A, Moussa M, Namdev N, Savage RE, Szwaya J, Volckova E, Westlund N, Wu H, Schwartz B. Discovery of 3-(3-(4-(1-Aminocyclobutyl)phenyl)-5-phenyl-3H-imidazo 4,5-b pyridin-2-y l)pyridin-2-amine (ARQ 092): An Orally Bioavailable, Selective, and Potent Allosteric AKT Inhibitor. Journal of Medicinal Chemistry, 2016; 59: 6455-6469.

[352] Weisner J, Landel I, Reintjes C, Uhlenbrock N, Trajkovic-Arsic M, Dienstbier N, Hardick J, Ladigan S, Lindemann M, Smith S, Quambusch L, Scheinpflug R, Depta L, Gontla R, Unger A, Muller H, Baumann M, Schultz-Fademrecht C, Gunther G, Maghnouj A, Muller MP, Pohl M, Teschendorf C, Wolters H, Viebahn R, Tannapfel A, Uhl W, Hengstler JG, Hahn SA, Siveke JT, Rauh D. Preclinical Efficacy of Covalent-Allosteric AKT Inhibitor Borussertib in Combination with Trametinib in KRAS-Mutant Pancreatic and Colorectal Cancer. Cancer Research, 2019; 79: 2367-2378.

[353] Uhlenbrock N, Smith S, Weisner J, Landel I, Lindemann M, Le TA, Hardick J, Gontla R, Scheinpflug R, Czodrowski P, Janning P, Depta L, Quambusch L, Muller MP, Engels B, Rauh D. Structural and chemical insights into the covalent-allosteric inhibition of the protein kinase Akt. Chemical Science, 2019; 10: 3573-3585.

[354] Quambusch L, Landel I, Depta L, Weisner J, Uhlenbrock N, Muller MP, Glanemann F, Althoff K, Siveke JT, Rauh D. Covalent-Allosteric Inhibitors to Achieve Akt Isoform-Selectivity. Angewandte Chemie-International Edition, 2019; 58: 18823-18829.

[355] Rai A, Oprisko A, Campos J, Fu YX, Friese T, Itzen A, Goody RS, Gazdag EM, Muller MP. bMERB domains are bivalent Rab8 family effectors evolved by gene duplication. Elife, 2016; 5.

[356] Bodor A, Radnai L, Hetenyi C, Rapali P, Lang A, Kover KE, Perczel A, Wahlgren WY, Katona G, Nyitray L. DYNLL2 Dynein Light Chain Binds to an Extended Linear Motif of Myosin 5a Tail That Has Structural Plasticity. Biochemistry, 2014; 53: 7107-7122.

[357] Nascimento AFZ, Trindade DM, Tonoli CCC, de Giuseppe PO, Assis LHP, Honorato RV, de Oliveira PSL, Mahajan P, Burgess-Brown NA, von Delft F, Larson RE, Murakami MT. Structural Insights into Functional Overlapping and Differentiation among Myosin V Motors. Journal of Biological Chemistry, 2013; 288: 34131-34145.

[358] Pylypenko O, Attanda W, Gauquelin C, Lahmani M, Coulibaly D, Baron B, Hoos S, Titus MA, England P, Houdusse AM. Structural basis of myosin V Rab GTPase-dependent cargo recognition. Proceedings of the National Academy of Sciences of the United States of America, 2013; 110: 20443-20448.

[359] Pylypenko O, Welz T, Tittel J, Kollmar M, Chardon F, Malherbe G, Weiss S, Michel CIL, Samol-Wolf A, Grasskamp AT, Hume A, Goud B, Baron B, England P, Titus MA, Schwille P, Weidemann T, Houdusse A, Kerkhoff E. Coordinated recruitment of Spir actin nucleators and myosin V motors to Rab11 vesicle membranes. Elife, 2016; 5.

[360] Klejnot M, Gabrielsen M, Cameron J, Mleczak A, Talapatra SK, Kozielski F, Pannifer A, Olson MF. Analysis of the human cofilin 1 structure reveals conformational changes required for actin binding. Acta Crystallographica Section D-Structural Biology, 2013; 69: 1780-1788.

[361] Hamill S, Lou HJ, Turk BE, Boggon TJ. Structural Basis for Noncanonical Substrate Recognition of Cofilin/ADF Proteins by LIM Kinases. Molecular Cell, 2016; 62: 397-408.

[362] Zimmet A, Van Eeuwen T, Boczkowska M, Rebowski G, Murakami K, Dominguez R. Cryo-EM structure of NPF-bound human Arp2/3 complex and activation mechanism. Science Advances, 2020; 6.

[363] von Loeffelholz O, Purkiss A, Cao LY, Kjaer S, Kogata N, Romet-Lemonne G, Way M, Moores CA. Cryo-EM of human Arp2/3 complexes provides structural insights into actin nucleation modulation by ARPC5 isoforms. Biology Open, 2020; 9.

[364] Prehna G, Stebbins CE. A Rac1-GDP trimer complex binds zinc with tetrahedral and octahedral coordination, displacing magnesium. Acta Crystallographica Section D-Structural Biology, 2007; 63: 628-635.

[365] Wurtele M, Wolf E, Pederson KJ, Buchwald G, Ahmadian MR, Barbieri JT, Wittinghofer A. How the Pseudomonas aeruginosa ExoS toxin downregulates Rac. Nature Structural Biology, 2001; 8: 23-26.

[366] Prehna G, Ivanov MI, Bliska JB, Stebbins CE. Yersinia virulence depends on mimicry of host Rho-family nucleotide dissociation inhibitors. Cell, 2006; 126: 869-880.

[367] Ferrandez Y, Zhang WH, Peurois F, Akendengue L, Blangy A, Zeghouf M, Cherfils J. Allosteric inhibition of the guanine nucleotide exchange factor DOCK5 by a small molecule. Scientific Reports, 2017; 7.

[368] Freedman SJ, Song HK, Xu YW, Sun ZYJ, Eck MJ. Homotetrameric structure of the SNAP-23 N-terminal coiled-coil domain. Journal of Biological Chemistry, 2003; 278: 13462-13467.

[369] Masuyer G, Stancombe P, Chaddock JA, Acharya KR. Structures of engineered Clostridium botulinum neurotoxin derivatives. Acta Crystallographica Section F-Structural Biology Communications, 2011; 67: 1466-1472.

[370] Agarwal R, Schmidt JJ, Stafford RG, Swaminathan S. Mode of VAMP substrate recognition and inhibition of Clostridium botulinum neurotoxin F. Nature Structural & Molecular Biology, 2009; 16: 789-U127.

[371] Kummel D, Krishnakumar SS, Radoff DT, Li F, Giraudo CG, Pincet F, Rothman JE, Reinisch KM. Complexin cross-links prefusion SNAREs into a zigzag array. Nature Structural & Molecular Biology, 2011; 18: 927-U1603.
